# Incorporation of Organic Counter‐Cations Into Poly(Heptazine Imide) Networks for Promoting Proton‐Coupled Electron Transfer During Photocatalytic H_2_O_2_ Evolution

**DOI:** 10.1002/anie.202524638

**Published:** 2026-06-13

**Authors:** Dingqiao Ji, Hikmat Binyaminov, Desiree Leistenschneider, Martin Oschatz, Paolo Giusto, Markus Antonietti, Horațiu Szalad

**Affiliations:** ^1^ Department of Colloid Chemistry Max Planck Institute of Colloids and Interfaces, Research Campus Golm Potsdam Germany; ^2^ Theoretical Bio‐ and Soft Matter Physics Freie Universitat Berlin Berlin Germany; ^3^ Institute For Technical and Environmental Chemistry Friedrich Schiller University Jena Jena Germany; ^4^ Center For Energy and Environmental Chemistry Jena (CEEC Jena) Friedrich Schiller University Jena Jena Germany; ^5^ Helmholtz Institute for Polymers in Energy Applications Jena (HIPOLE Jena) Jena Germany

**Keywords:** cation exchange, ionic carbon nitride, organic counter‐cations, photocatalytic H_2_O_2_ evolution, poly(heptazine imide)

## Abstract

Nitrogen based onium ions such as methylammonium (MA^+^), formamidinium (FA^+^), and tetramethylammonium (TMA^+^) were used as counterions in poly(heptazine imide) (PHI) networks via a protonation‐acid base reaction strategy. This creates all organic PHIs. Chemical composition analysis confirmed the successful accommodation of target species, resulting in a maximum incorporation of 1 cation per ideal PHI unit cell. Structural characterization confirmed crystal phase consistency to that of parent Na‐PHI. Photocatalytic H_2_O_2_ evolution was chosen as a model reaction. Under ambient conditions, MA^+^‐PHI showed the best performance, producing 7.6 mmol g^−^^1^ H_2_O_2_ after 1 h of 427 nm LED irradiation, corresponding to an AQY of 13.9% and a 42% activity enhancement over the Na‐PHI analogue. When an additional oxygen pressure of 3 bars is applied, the H_2_O_2_ yield increased to 36.5 mmol g^−^^1^, while AQY increased proportionally to ∼66.8%, being one of highest outputs reported to date. Photochemical and photo‐charging experiments suggest that methylammonium moieties stabilize photoelectrons though coulombic interaction in an effective manner, while proton conductivity and photocatalytic experiments point out toward a PCET driven mechanism responsible for the recorded H_2_O_2_ rate enhancements. These results showcase that counterion replacement, as applied in organic perovskites, also improves carbon nitride photosynthesis.

## Graphical abstract

Incorporation of N‐based onium ions into the ionic framework of poly(heptazine imide) was obtained via protonation‐deprotonation of PHI frameworks arising from a parent Na‐containing analogue. Particularly, methylammonium moieties, through their small molecular size, hydrogen bonding capabilities and rotational mobility, appear to stabilize photogenerated electrons via coulombic pair formation, while also facilitating a PCET mechanism during H_2_O_2_ evolution.

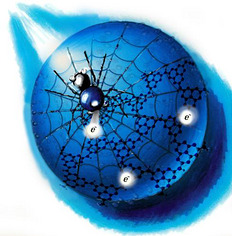

## 1 Introduction

In recent decades, photocatalysis has emerged as a key strategy for addressing global challenges related to energy and environmental sustainability. By harnessing solar irradiation, a clean and abundant energy source, photocatalytic processes enable the conversion of light into chemical energy via various redox processes [1], some notable examples being water splitting, CO_2_ reduction, H_2_O_2_ evolution, among others. Early research on catalytic solar transducers focused largely on metal oxides [2] (e.g., TiO_2_, ZnO) and metal sulfides [3] (e.g., CdS). However, such photocatalytic systems are still to overcome the three main bottlenecks associated with solar driven chemical processes, mainly poor spectral response in the visible region, fast electron‐hole recombination and limited thermodynamic capabilities [4]. These drawbacks hinder technology deployment of large‐scale photocatalytic processes.

In search of alternatives, research interest shifted toward metal‐free photocatalysts, particularly those composed of light, earth‐abundant elements such as carbon and nitrogen. Within this domain, polymeric carbon nitride (PCN) stands out due to its metal‐free composition, relatively facile synthesis, and a narrower bandgap, which allows photoactivity under visible‐light. Moreover, the polymer‐like, adaptable structure of carbon nitride offers opportunities for tuning its electronic and surface properties through doping, templating, and other chemical modifications [5]. Poly(heptazine imide) (PHI) is a widely studied carbon nitride based material, primarily because of its outstanding performance in various photocatalytic reactions, which can be partly attributed to its higher polarizability and a lower charge carrier recombination rate compared to conventional graphitic carbon nitride [6]. Owing to its straightforward synthesis, numerous studies have focused on modifying its microstructure.

Incorporation of molecular moieties into PHI networks is an attractive synthetic strategy that carries potential benefits such as the introduction of advanced donor‐acceptor systems with modulated π–π* and n‐ π* electronic transitions [7], modulated adsorption modes of substrates via molecular interactions with accommodated guest moieties [5, 8], incorporation of proton relays to improve PCET driven reactions [9] and the introduction of catalytically active centers [10]. Through co‐polymerization of different C and N bearing substrates such units can be introduced, a notable example reporting melem and benzene‐1,4‐dicarboxylic acid dihydrazide as precursors for PHI networks which exhibited photocatalytic NH_3_ outputs of 83.3 µmol g^−1^ h^−1^ and AQYs reaching 2.31% (420 nm) [11]. Post‐synthetic approaches involve either the functionalization of preformed PHI networks through existing functional groups, or make use of N‐rich structural channels for the electrostatic anchoring of said moieties. One such example employs a dicyandiamide post‐treatment that yields a melamine functionalized PHI structure with HER outputs reaching 5.6 mmol g^−1^ h^−1^ (TEOA and 1.75 wt.% Pt) [12]. Another example introduces Zn^2+^ coordination noted into the PHI network, and thus, meso‐tetrakis(4‐carboxyphenyl)porphyrin could be anchored, resulting in a catalyst with notable activity in the photocatalytic OWS reaction (1.06 mmol g^−1^ of H_2_ in 12 h under white light irradiation) [13].

Hybrid perovskites, compared to their purely inorganic analogues, have been successfully employed as solar transducers for highly efficient photon conversion into electric energy. Outstanding photovoltaic performances were described to arise from the incorporation of organic cations such as methylammonium (MA^+^) [14] or formamidinium (FA^+^) [15] in‐between metal‐halide octahedra (MX_6_). Due to their small molecular size, hydrogen bonding capabilities and rotational mobility [16], the carrier lifetime is greatly improved. However, despite their exceptional photophysical properties, hybrid perovskites are unsuitable candidates for photocatalytic technologies due to their poor chemical stability, and potential toxicity of certain metal components [17]. Similarly, substituting metallic counterions in PHI with onium ions may offer comparable benefits (enhancing or modifying charge transport properties). Locally building on the idea of substituting metal cations with organic cations, methylammonium (MA^+^) and formamidinium (FA^+^) offer a particularly interesting case for enhancing proton conductivity. Unlike purely inorganic cations, MA^+^ and FA^+^ molecules include mobile protons and can undergo interaction with delocalized protons, thereby creating an efficient pathway for proton shuttling. In contrast, metal cations in PHI typically lack such extensive hydrogen‐relays. Recent COF studies further highlight that subtle molecular‐structure choices can simultaneously improve activity and robustness: linkage‐position control in COFs enabled sustained, sacrificial‐reagent‐free H_2_O_2_ photosynthesis in pure water for up to 48 h, underscoring the importance of local chemical environment in stabilizing productive reaction pathways [18].

The previous development of metal ion free organic‐cation‐based PHI remains very limited. One previous work tackling ion exchange in PHI networks reported that tetramethylammonium (TMA^+^) could not replace the K^+^ ions originally present in the framework, although other cations, such as Na^+^ and Cs^+^, were successfully introduced under similar condition [19]. Larger ionic radius of TMA^+^ (approximately 3 Å) could be the key factor that inhibit the ion exchange. Thus, ionic conductivity values reported for this sample (8·10^−8^ S cm^−1^) does not reflect the actual potential of PHI networks incorporating organic cations.

This study proposes an alternative approach that enables a broad range of cation substitutions, with nitrogen‐based onium ions as a main focus. H‐PHI, a completely covalent poly(heptazine imide) network, was treated with DBU (1,8‐Diazabicyclo[5.4.0]undec‐7‐ene), a strong organic base with a molecular size that cannot enter the PHI channels due to size misfit, and which compensates for the weak alkalinity of MA^+^, FA^+^, and TMA^+^ salts. Thus, complete deprotonation of the PHI network is achieved, facilitating the incorporation of co‐dissolved onium ion salts. Photocatalytic O_2_ reduction to H_2_O_2_ has been chosen as a suitable reaction model for probing the effect of aforementioned cations on the activity of PHI. Herein, MA^+^‐PHI was found to be 42% more active than the initial Na‐PHI in the studied reaction, reaching H_2_O_2_ production rates of 7.6 mmol g^−1^ h^−1^ under visible light irradiation (427 nm). Moreover, under Ar atmosphere, MA^+^‐PHI shows faster electron storing properties (0.55 mmol e^−^·h^−1^) compared to Na‐PHI (0.36 mmol e^−^·h^−1^), which in conjunction with EPR and ionic conductivity measurements highlight the advantages of organic cation incorporation, and the successful embedding of perovskite‐like mechanistic behaviors into PHI photocatalysts.

## 2 Results and Discussion

**SCHEME 1:**
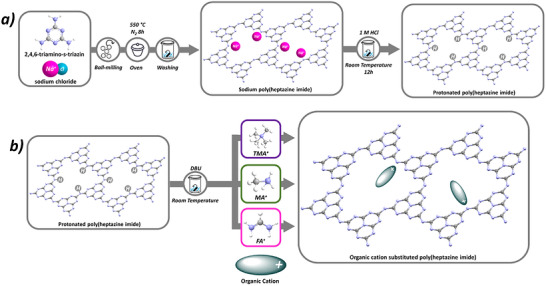
Illustration of the synthetic procedure adopted for obtaining the Na‐PHI and H‐PHI matrixes, (a) which were thereafter further processed so that the synthesis of target Cat^+^‐PHI photocatalysts could be achieved (b).

A set of three PHI matrixes incorporating onium ions (TMA^+^, MA^+^, and FA^+^) were synthesized by employ of a stepwise procedure described both illustratively in Scheme 1, and with further detail in the supplementary materials (**S.1.1**). In a first step, the Na‐PHI framework was yielded through the polycondensation of a melamine precursor at 550°C in the presence of NaCl [20], structure which was thereafter desodiated and exchanged with hydrogen via acidic treatment (1 M HCl) [21]. To allow the incorporation of target cations, this metal‐free H‐PHI network was deprotonated in the presence DBU, a strong organic base, which allowed the reintroduction of ionic character into the PHI structure, and the stabilization of said organic cations into the six‐ring heptazine channels structurally illustrated in Scheme 1. Thus, this synthetic strategy has yielded a completely organic, ionic PHI structure, which to our knowledge is the first successful attempt to date.

**FIGURE 1:**
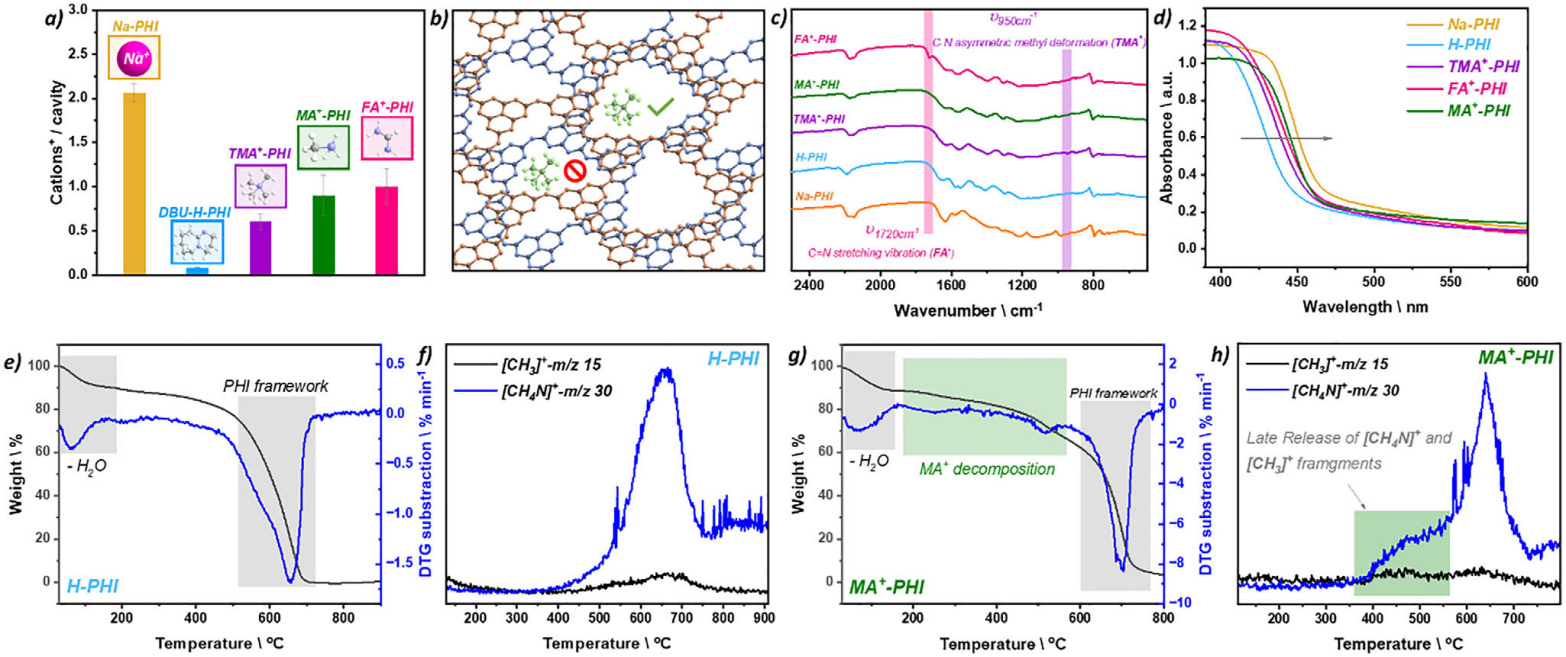
Cation content per ideal PHI lattice for samples under study. Na^+^ content was determined via ICP‐OES measurements, while incorporation of onium cations was calculated from N:C ratios recorded via EA (a); illustrative description of organic cation accommodation into PHI structures with rotational stacking defects, TMA^+^ being chosen as an example due to large ionic radii (b); FTIR spectra recorded for the samples under study (c); DR UV–vis spectra acquired for investigated PHI specimens (d); TGA decomposition with recorded DTA signals for H‐PHI (e) and MA^+^‐PHI (g); recorded MS surveys during thermal decomposition of H‐PHI (f) and MA^+^‐PHI (h)

In order to quantify the amount of organic cation accommodated in the PHI network for each respective case, N:C ratios were measured via elemental analysis (Table S1). Na‐PHI and H‐PHI both yielded ratios of approximately 1.41, all deviations from this standard value being considered as a compositional contribution arising from incorporated organic cations. To be noted that a small deviation could also be observed for H‐PHI after DBU treatment, which we attribute to surface adsorption of said molecules. Thus, Figure 1a describes the number of cations accommodated in a perfect PHI cavity. The Na‐PHI analogue exhibits around 2 Na^+^ per cavity (ICP‐OES), while TMA^+^‐PHI, MA^+^‐PHI and FA^+^‐PHI show 0.6, 0.9 and 1 cations of their respective species. Low TMA^+^ loadings are to be attributed to their large ionic radii (approximately 3 Å), compared to FA^+^ (approximately 2.5 Å) and MA^+^ (around 2.2 Å) [22]. The molecular structure of poly(heptazine) imide is best described as layers containing an array of equivalent equilateral triangular nanocavities (height of approx. 1.1 nm), which in an ideal case would align above one another (AAA‐type stacking), yielding a continuous triangular prism channel through the stacks [6]. In practice, experimental specimens of PHI exhibit a layer stacking which doesn't follow a strict registry. Instead, layers are stacked with a small angle of rotation between each other [23]. Such geometrical misalignments have practical consequences for cation uptake. Although the TMA^+^ cation is nominally smaller than the ideal triangular pore, the overlapping region between pores of adjacent layers become a smaller polygon. Thus, not all such apertures can be freely thread by onium cations (Figure 1b).

Organic cation incorporation has also been probed via ^1^H‐NMR measurements of solution aliquots before and after addition of DBU (Figure S1; Table S2). The decrease in peak area was monitored at 3.3, 2.25 and 8 ppm for TMA^+^, MA^+^, and FA^+^ respectively, and integrated to the internal standard. Cation contents of 0.73, 1.27 and 0.94 (per ideal lattice) were therefore calculated. The slight discrepancy observed in the case of TMA^+^ and MA^+^ may arise from surface adsorption of said species, which in the standardized procedure would be removed via washing, yet calculated values obtained through both EA and NMR are consistent.

FTIR analysis of all PHI samples under study (Figure 1c) show specific vibrational spectra characteristic to heptazine networks, with distinct signals observed in the 800–1650 cm^−1^ region [24]. Notably, TMA^+^‐PHI exhibits an additional vibration band at 950 cm^−1^, which is attributed to the C–N asymmetric methyl deformation characteristic to the tetramethylammonium guest [25], while FA^+^‐PHI shows an addition band at 1720 cm^−1^ corresponding to C = N asymmetric stretching vibrations of formamidinium [26]. Although C–N and C = N linkages are also intrinsic to the PHI scaffold, the local bonding environments are fundamentally different in the polymeric heptazine lattice versus the molecular onium cations. Therefore, guest‐specific vibrations can appear as additional absorptions (and/or as changes in band position/linewidth) even within the same nominal functional‐group region. In the case of MA^+^‐PHI, no distinct methylammonium‐related band is resolved, which is expected because the C─N single‐bond vibration of MA^+^ overlaps strongly with the dense C─N stretching manifold of the PHI framework. Because TMA^+^ (N(CH_3_)_4_^+^) is close to tetrahedral symmetry, it exhibits a limited set of strong, well‐defined IR‐active modes (often giving sharp CH_3_ deformation/C‐N‐associated bands), whereas MA^+^ (CH_3_NH_3_^+^) has lower symmetry and its vibrations couple strongly to NH_3_ motions and hydrogen bonding, leading to more distributed and broadened absorptions and hence invisible in this case [27]. It must be noted that all samples under study exhibit a vibration mode at 2160 cm^−1^ associated with nitrile end‐groups decorating the PHI surface, indicating that the harsh chemical processing during framework desodiation and deprotonation does not significantly alter the polymer surface. Additionally, indirect evidence of cation incorporation can be drawn from acquired UV–vis DR spectra (Figure 1d), considering the absorption onsets of each individual sample. After acidic treatment and desodiation of Na‐PHI, a blue‐shift from 463 to 446 nm is recorded. Upon cation reincorporation, absorption contributions redshift toward this initially recorded value, TMA^+^‐PHI, MA^+^‐PHI, and FA^+^‐PHI exhibiting onsets at 457, 461, and 460 nm, respectively. Such phenomenology has been previously described, where reincorporation of K^+^ ions in a K‐PHI derived protonated sample would lead to a recovery of initial UV–vis contribution [21]. This reverse shift indicates molecular structure recovery. On the other hand, if onium ions form clusters or undergo molecular grafting, such a backwards redshift would not be observed.

TG‐DTA and TG‐MS were carried out for further confirmation of onium cations embedded into the PHI network. The parent H‐PHI sample (Figure 1e) exhibits a two‐step decomposition behavior, with initial weight losses between 35 and 175°C derived from moisture release, followed by PHI framework collapse starting at 500°C, with release of [CH_3_]^+^ and [CH_4_N]^+^ (Figure 1f) as well as [CO_2_]^+^ mass fragments. TMA^+^‐PHI, MA^+^‐PHI, and FA^+^‐PHI exhibit similar decomposition patterns (Figures 1g and S2), with additional losses between 200 and 500°C arising from TMA^+^ ([CH_3_]^+^, [CH_4_N]^+^, [C_2_H_2_N]^+^, [C_2_H_4_N]^+^, [C_3_H_8_N]^+^, [C_3_H_9_N]^+^), and FA^+^ ([CH_3_]^+^, [CH_4_N]^+^) thermal decomposition and release (Figure S3). Notably, compared to its two analogues, MA^+^‐PHI does not show any low temperature release (between 200–300°C) of characteristic [CH_3_]^+^ or [CH_4_N]^+^ mass fragments, emission of such species starting only after 400°C (Figure 1h). Such a late discharge of methylammonium‐derived species factors both the conformational flexibility of these molecular guests and H‐bonding with acceptor sites structurally present in the PHI triangular cavity, behavior previously observed in a MAPbI_3_ hybrid perovskite [28]. MA^+^ has N–H donors and can form directional H‐bonds with PHI acceptor sites (ring N, deprotonated imide N) and interlayer water. This stabilizes local MA^+^···N(PHI) contacts and promotes formation of ion pairs/clusters. This makes MA^+^‐PHI more stable than methylammonium chloride, which would decompose at around 250°C.

**FIGURE 2:**
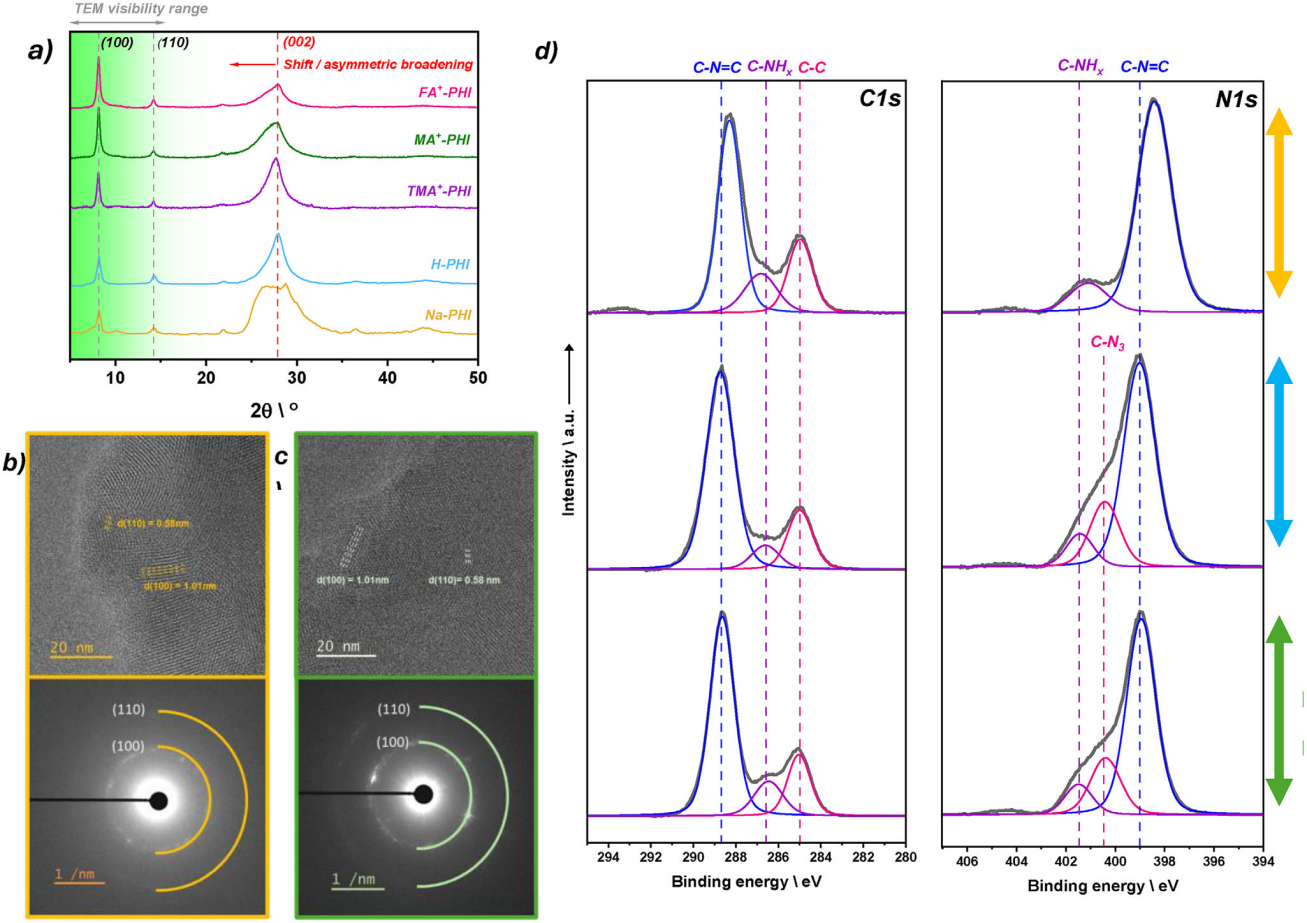
X‐ray diffraction patterns recorded for all investigated PHI samples, with specific diffraction lines being indexed at their respective positions (a); HR‐TEM images and SAED patterns acquired for Na‐PHI (b) and MA^+^‐PHI (c); deconvoluted C1s (d) and N1s (e) XPS spectra of Na‐PHI, H‐PHI, and MA^+^‐PHI, with each signal assigned to a specific chemical environment

Powder x‐ray diffraction patterns recorded for all studied samples (Figure 2a) show the characteristic PHI reflections at 8.2° and 14.2° (2θ), assigned to the (100) and (110) planes, respectively. The diffraction region around 25°–29° is commonly discussed as the “(002) graphitic reflection” (∼28°), yet for PHI it also contains overlapping in‐plane contributions. Our PXRD simulations (Figure S4) indicate that the (220), (101), and (300) reflections fall within this range and therefore co‐shape the apparent peak profile [6]. In Na‐PHI, the reduced sharpness/intensity at ∼28° is thus not indicative of poor layer stacking, but rather reflects a stronger contribution of these in‐plane components and their sensitivity to crystallinity in the (a,b) plane: when the in‐plane order decreases (as in H‐PHI and the cation‐exchanged derivatives), the (220)/(101)/(310) components weaken while the (002) contribution remains essentially unchanged, which visually “sharpens” the feature into a more intense peak centered near ∼28°. Consistent with this interpretation, H‐PHI and the derived materials exhibit a sharper, more intense (002)‐dominated reflection indicative of more uniform interlayer periodicity, whereas Na‐PHI retains a broader composite feature. Upon exchanging protons for larger organic cations, the ∼28° feature further broadens toward lower angles, indicating local curvature and a distribution of interlayer spacings introduced by steric strain from bulkier ions. Importantly, the average basal spacing associated with the (002) planes remain remarkably constant at 0.32 ± 0.01 nm across all PHI variants (deviation < 5%) indicating that bulk ion exchange does not destruct the fundamental heptazine framework. HR‐TEM images and SAED patterns of both Na‐PHI (Figure 2b) and MA^+^‐PHI (Figure 2c) allow to distinguish between the (100) and (110) domains. No satellite reflections, or additional lattice spacings are observed, confirming single‐phase integrity. Despite this, it must be noted that PHI desodiation and further cation incorporation induce morphological changes in bulk (Figure S5).

Surface chemical analysis was carried out via XPS, the C1s spectra of Na‐PHI, H‐PHI and MA^+^‐PHI (Figure 2d) showing three main components associated with different types of carbon environments, mainly C─N = C (288.2–288.7 eV) and C─NH*_x_* (286.4–286.7 eV) derived from the heptazine network and C‐C (284.9 eV) originating from adventitious carbon [29, 30]. Notably, after acidic treatment of the parent Na‐PHI sample, the C─N = C peak of H‐PHI and MA^+^‐PHI showed a 0.5 eV shift toward higher binding energies, which can be attributed to a decrease in the anionic character of the PHI network, as well as surface oxidation which is indicated by the O1s spectra recorded for the same specimens (Figure S5). Moreover, no significant Na 1s signal can be observed in the acid treated PHI, while the spectra of Na‐PHI is shown in Figure S6. Contributions arising from the methyl groups of MA^+^ can be seen for the C─NH*_x_* peak at 286.4 eV [31] where both aperture and peak area ratios between C─N = C and C─NH*_x_* (8.3 for H‐PHI and 4.9 for MA^+^‐PHI) indicate a higher population of such species. N1s spectra of investigated samples (Figure 2e) show three main peaks, C─N = C (398.7–399) and C─NH*_x_* (401–401.2 eV) for all samples under study, while the C─N_3_ (400.4 eV) peak could be clearly defined for H‐PHI and MA^+^‐PHI. Similar shifting behavior could be seen for C─N = C contributions in Na‐PHI and H/MA^+^‐PHI in the C1s spectra, while recorded peak area ratios between C─N = C and C─NH*_x_* signals for H‐PHI and MA^+^‐PHI were 7.2 and 6.9, respectively.

To examine whether methylammonium incorporation is structurally compatible with the PHI lattice, DFT calculations were performed on an MA^+^–H–PHI model in a hexagonal unit cell. Additional technical information is provided in the supporting Information. The optimized cell showed only a small deviation from the parent PHI lattice, indicating that the heptazine framework remains essentially rigid after MA^+^ incorporation. To find the nontrivial coadsorption sites and coorientations of two multiatom MA^+^ groups, as well as the proton binding site, a systematic configurational search was carried out (full set in Table S3, starting positions in Figure S7b), and the four lowest‐energy structures (two lowest per each tested protonation site) are summarized in Figure S8. After geometry optimization, thermal annealing, and final relaxation, all stable solutions converged to motifs in which the ammonium end of MA^+^ is directed toward PHI nitrogen sites, consistent with stabilizing hydrogen‐bonding interactions. Together with the experimentally preserved PHI phase and recovered structural/spectral features discussed above, these results show that the DFT‐calculated stable structure is consistent with the predicted PHI structure.

DFT calculations identified four low‐energy configurations for MA–H–PHI, namely A‐BC‐cis, A‐BC‐trans, F‐AC‐cis, and F‐AC‐trans (Figure S8). In all optimized structures, the two MA^+^ cations occupy corner positions in the PHI cavity, with their ammonium ends directed toward framework nitrogen atoms and spatially separated from the proton. The cations adopt cis or trans out‐of‐plane arrangements, with only a marginal energy difference with the proton at site A (0.8 kJ mol^−^^1^), but a clear preference for the trans isomer with the proton at site F (6 kJ mol^−^^1^). Among these structures, A‐BC‐trans is 18 kJ mol^−^^1^ more stable than F‐AC‐trans, although the experimentally accessible protonation state may ultimately determine which configuration is experimentally relevant. Acquired band structures and total density of states (DOS) for the four described MA^+^‐PHI configuration are illustrated in Figure S9, with acquired band gaps ranging between 2.1–2.3 eV, denoting a consistent electronic structure between conformers. We note that these bandgap values are calculated for monolayers at a certain level of theory, which is known to significantly underpredict the gaps. Therefore, only qualitative trends should be compared to the experiments. Calculated Bader charges (Tables S4 and S5) show a net charge of +0.8 rather than a formal +1.0 on each MA^+^ group (irrespective of the configuration), indicating a transfer of electron density from the PHI core back into the MA^+^ moieties. This charge transfer specifically targets the highly descreened ammonium protons, leaving the adjacent methyl protons relatively non‐polar. This combination of fractional intermolecular charge transfer and localized proton descreening serves as the topological signature of strong hydrogen bonding between the PHI nitrogens and the MA^+^ units. Comparisons across different structural isomers confirm that this robust hydrogen‐bonding network dictates the electronic state, rendering the overall charge distribution virtually immune to changes in the geometric orientation of the MA^+^ groups.

In order to probe the effect of onium ion incorporation onto the photocatalytic properties of PHI materials, H_2_O_2_ evolution has been chosen as model reaction. Standard photocatalytic experiments were carried out in gas‐tight glass reactors, under saturated O_2_ atmosphere, with a 427 nm LED (0.1 W cm^−2^) as the irradiation source. A detailed experimental procedure is further described in the supplementary material.

**FIGURE 3:**
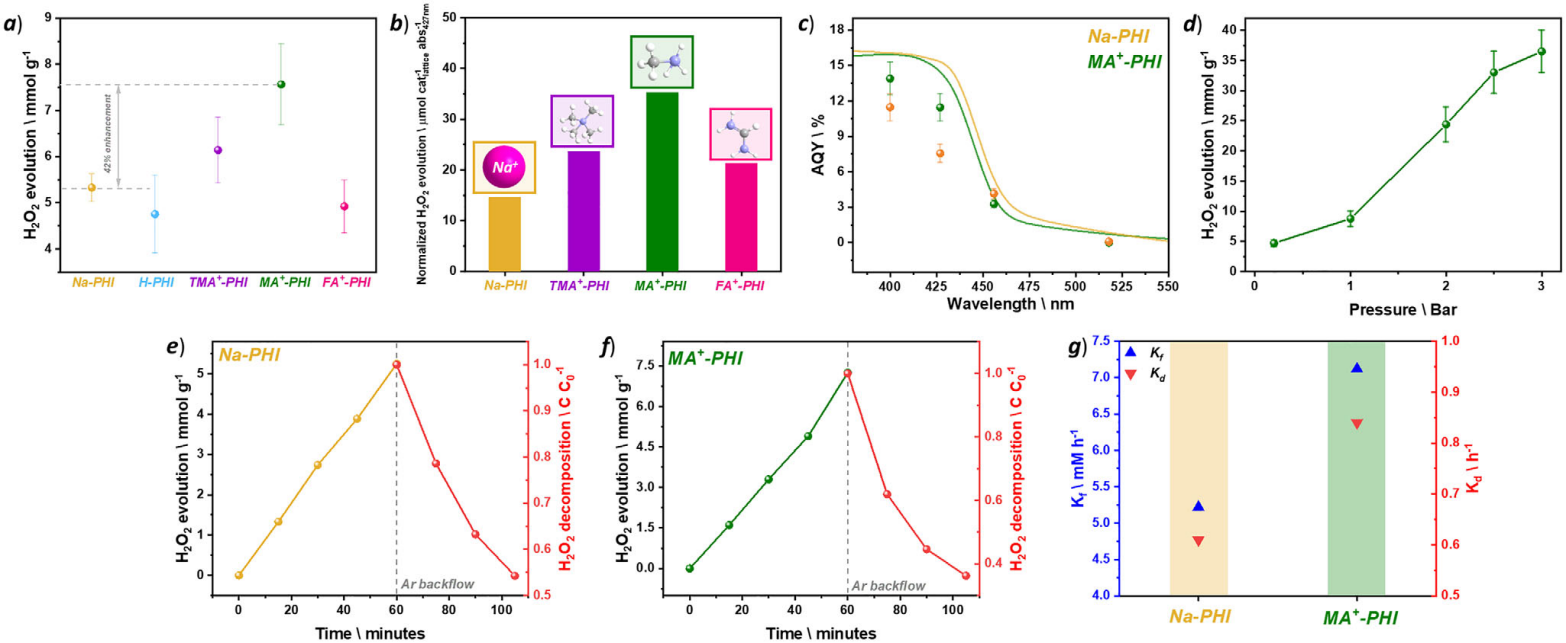
Photocatalytic screening of samples under study for H_2_O_2_ evolution, all reactions being carried out for one hour in a 10 v.% glycerol solution under irradiation from a 427 nm LED (a); H_2_O_2_ photocatalytic yield normalized by cation incorporation and spectral response (b); photo‐action spectra of Na‐PHI (yellow balls) and MA^+^‐PHI (green balls) carried out under no O_2_ overpressure, with DR UV–vis spectra included in the background (Na‐PHI yellow lines and MA^+^‐PHI green lines) (c); O_2_ pressure control tests carried out for MA^+^‐PHI (d); kinetic curves for both H_2_O_2_ formation and decomposition for the Na‐PHI (e) and MA^+^‐PHI (f) photocatalysts; the K_f_ (blue) and K_d_ (red) values calculated for Na‐PHI (yellow) and MA^+^‐PHI (green) (g)

The H_2_O_2_ output of herein synthesized PHI photocatalysts was assessed after one hour (Figure 3a), clear differences are observed as function of cation accommodated in the polymeric network. The parent Na‐PHI sample shows an H_2_O_2_ yield of 5.3 mmol g^−1^, while H‐PHI derived from the same specimen had a lower photocatalytic performance (4.7 mmol g^−1^ H_2_O_2_ under same conditions). Factoring to this decrease are changes in spectral response described in Figure 1d, as well as physico‐chemical alterations, as seen in the XPS survey and previously described in literature [21]. Onium cation modification resulted in significant H_2_O_2_ yield enhancements (as compared to parent Na‐PHI) for TMA^+^‐PHI and MA^+^‐PHI, with 6.2 (17%) and 7.6 (42%) mmol g^−1^, respectively. FA^+^‐PHI on the other hand exhibited a decrease in photocatalytic activity (4.9 mmol g^−1^) under employed conditions. In order to isolate the performance effect derived from TMA^+^, MA^+^, and FA^+^ inclusion in PHI structures, variations such as mean cation content and light harvesting properties (specifically for the experimental 427 nm LED source) need to be considered. Thus, a normalization parameter accounting for spectral response of each photocatalysts was calculated and listed in Table S6 (methodology described in the Supporting Information), while cation content was previously discussed in Table S1. Normalized H_2_O_2_ outputs are compared in Figure 2b. The contribution of Na^+^ ions in Na‐PHI would therefore account for 14.5 µmol cat_lattice_^−1^·abs_427_^−1^, while enhanced photocatalytic properties arise by individual cation moiety, with 23.7, 35.3 and 21.3 µmol cat_lattice_^−1^·abs_427_^−1^ obtained for TMA^+^, MA^+^ and FA^+^, respectively. Products evolving via the oxidative half‐reaction of glycerol were further tested for MA^+^‐PHI. Liquid‐phase quantitative ^1^H NMR analysis of the post‐reaction solution reveals that glycerol oxidation predominantly yields formic acid (∼70%) and lactic acid (∼30%), quantified by peak intensity integration (Figures S10 and S11). These products indicate that, under our conditions, glycerol undergoes substantial oxidative transformation, including C─C bond cleavage leading to C1 products (formate/formic acid) and a parallel pathway yielding a C3 hydroxy‐acid (lactic acid). Apart from these two species, glycerol oxidation into glycolic acid might also occur, but the peak at 4.3 cannot be quantified due to the relatively low intensity and is influenced by the water specific peak. The formation of C3 oxygenates such as dihydroxyacetone (DHA) is frequently observed in PHI‐driven glycerol valorization and has been reported as a selective product in fully condensed potassium PHI systems, and DHA (or related C3 intermediates) is also recognized as a key precursor relevant to lactic‐acid‐forming routes. The dominance of formic acid in our system is consistent with photocatalytic mechanisms in which photogenerated holes and superoxide species promote glycerol oxidation via C─C cleavage sequences toward formic acid. More broadly, glycerin oxidation has been demonstrated to couple with H_2_O_2_ production in advanced photocatalytic platforms, supporting that glycerin is actively oxidized in such dual‐functional systems [32, 33].

While most carbon nitride systems target H_2_O_2_ formation via the 2e^−^ oxygen reduction pathway, alternative oxidative routes are also possible. In a notable precedent, atomically dispersed Mn on carbon nitride has been shown to generate H_2_O_2_ through direct and indirect 2e^−^ water oxidation processes, emphasizing the need for pathway‐discriminating controls [34]. The photocatalytic evolution of H_2_O_2_ undergoes via the 2e^−^ reduction of O_2_, as proven by experiments carried out in the absence of either glycerol or oxygen (Ar purged system), which didn't yield any detectable amount of product. Thus, photogenerated h^+^ do not play a constructive role in the accumulation of H_2_O_2_, and need to be quenched via additives that therefore mitigate oxidative activation of the discussed product. The reductive and oxidative reaction pathways are described in Equations **1**–**7**. Upon photoexcitation of PHI materials, excitons are generated and further dissociate into e^−^ and h^+^ available for their respective half‐reactions. Photoelectrons populating the CB of the considered semiconductor are transferred to O_2_ molecules, yielding H_2_O_2_ (2e^−^ and 2H^+^). Through simultaneous transfer of electrons and protons (PCET), H_2_O_2_ evolution can be further improved, as reaction intermediates otherwise generated stepwise are susceptible to oxidative breakdown. On the other hand, photogenerated h^+^ are depleted via glycerol oxidation.

(1) $$MA^{+}-\mathrm{PHI}\overset{h\upsilon }{\to }\;MA^{+}-\mathrm{PH}I^{*}\left(e_{\mathrm{CB}}^{-}/h_{\mathrm{VB}}^{+}\right)$$

(2) $$O_{2}+e_{\mathrm{CB}}^{-}\to O_{2}^{\cdot -}$$

(3) $$O_{2}^{\cdot -}+H^{+}\to \mathrm{HO}O^{\cdot }$$

(4) $$\mathrm{HO}O^{\cdot }+e_{\mathrm{CB}}^{-}\to \mathrm{HO}O^{-}$$

(5) $$\mathrm{HO}O^{-}+H^{+}\to \mathrm{HOOH}$$

(6) $$\mathrm{Glycerol}+\;\;h_{\mathrm{VB}}^{+}\to \mathrm{Glycero}l_{\mathrm{ox}}$$

(7) $$\mathrm{HOOH}+2h_{\mathrm{VB}}^{+}\to O_{2}+2H^{+}$$

The described photo‐reactivity pathway has been further evidenced via in situ EPR measurement of oxygenated acetonitrile suspensions containing all PHI photocatalysts under investigation, while DMPO acted as spin‐trap agent. Spectra recorded before light irradiation did not show any response (Figure S12a), while photo‐irradiated samples exhibited EPR spectra showcasing lines between 3415 and 3455 G (Figure S12b) characteristic to DMPO‐^•^OOH adducts, therefore confirming the intermediacy of hydroperoxyl species.

As MA^+^‐PHI showcased the most notable photocatalytic enhancement as compared to the parent Na‐PHI analogue, further mechanistic investigation was carried out highlighting the effect of methyl ammonium inclusion into the PHI network. The photo‐action spectra of MA^+^‐PHI and Na‐PHI were recorded for wavelengths corresponding to visible light contributions, from 400 to 520 nm (Figure 3c), following the methodology described in the supplementary material, with associated incident light intensities listed in Table S7. Under each spectral contribution, H_2_O_2_ yields were acquired after one hour of photocatalytic output. It was observed that H_2_O_2_ generation and absorption spectra of both samples show a moderate interdependence, with AQY values gradually decreasing towards longer wavelengths. Thus, negligible photo‐response is recorded for 520 nm irradiation. Interestingly, a clear difference between Na‐PHI and MA^+^‐PHI is observed at 400 nm, with AQYs of 11.5 and 13.9% respectively. Furthermore, H_2_O_2_ yield enhancements arising from MA^+^ structural inclusion is even more apparent at 427 nm, with Na‐PHI showcasing an AQY of 7.6%, while 11.5% was recorded for MA^+^‐PHI. To further stress the applicability of this novel PHI synthetic approach for the herein described photocatalytic model, H_2_O_2_ evolution was carried out under an increment of O_2_ overpressures, ranging from atmospheric to 3 bars (Figure 3d). Therefore, recorded H_2_O_2_ yields highlight a proportional increment with O_2_ availability, resulting in a maximum performance of 36.5 mmol g^−^^1^, and an associated AQY of 66.8%, one of the highest recorded values to date. This high performing photocatalytic model was compared to analogous literature reports, a comprehensive report being listed in Table S8.

Additional correlations between methyl ammonium accommodation into PHI networks and the enhanced photocatalytic performance can be acquired by assessing the kinetic model of O_2_ reduction towards H_2_O_2_, comprising of both zero order (k_f_) and first order (k_d_) components, as described in Equation 8.

(8) $$\left[H_{2}O_{2}\right]=\frac{k_{f}}{k_{d}\left(1-e^{-k_{d}\cdot t}\right)}$$

In porous organic photocatalysts, net H_2_O_2_ accumulation can be strongly limited by photocatalyzed product decomposition, as for example, pyrene‐based COFs were shown to exhibit higher H_2_O_2_ loss when pyrene units are locally concentrated [35]. Therefore, formation and decomposition kinetics were assessed for both Na‐PHI and MA^+^‐PHI by first recording H_2_O_2_ yields at defined times, finally resulting in stationary H_2_O_2_ accumulations of 5.3 and 7.4 mmol g^−1^, for Na‐PHI and MA^+^‐PHI, respectively, after one hour of irradiation from a 427 nm LED. The O_2_ rich atmosphere inside the reactor was thereafter flushed via an argon flow, after which the reaction mixture was irradiated for an additional 45 min with solution aliquots being gradually recorded. H_2_O_2_ decompositions of 46% and 63% were therefore recorded for the Na and MA^+^ analogues (Figure 3e for Na‐PHI and Figure 3f for MA^+^‐PHI). By fitting acquired data to Equation 8, resulted k_f_ values showed a greater formation rate of H_2_O_2_ for M^+^‐PHI (7.12 mM h^−1^) than Na‐PHI (5.22 mM h^−1^), while k_d_ decomposition rates of 0.61 and 0.84 h^−1^ were recorded for the two photocatalysts (Figure 3g). Referencing from previous studies, thermal decomposition rate of H_2_O_2_ is negligible compare with photocatalyzed decomposition. It can therefore be concluded that enhanced photocatalytic H_2_O_2_ outputs observed for the methyl ammonium sample arise from greater formation rates, which compete overall with either oxidative or reductive activation of yielded product [36].

Recyclability of MA^+^‐PHI was later tested by acquiring H_2_O_2_ yields during a series of four consecutive catalytic experiments carried under identical conditions as previously described (10% glycerol, O_2_ atmosphere, 427 nm LED irradiation for one hour), the employed material being washed with ultrapure water after each run. H_2_O_2_ outputs decreased from an initial 7.6 to 6.4 mmol g^−1^ recorded after the fourth cycle, equivalent to a 16% drop (Figure S13). Despite this, recorded photocatalytic performances are still larger than that of Na‐PHI, and may be considered for even further operational times. To be noted that a MA^+^ leaching of approximately 20% could be observed after the first catalytic run (observed by ^1^H‐NMR), while no additional leaching could be observed for subsequent experiments. After catalytic cycling, the MA^+^‐PHI powder was collected, washed, and dried following the previously described procedure, and then characterized. The fresh and cycled materials show highly consistent characterization results. Minor differences are observed in the intensity of the C‐NH*_x_* signal in the C 1s XPS spectrum, suggesting slight leakage of methylammonium (around 20%), whereas no identifiable difference is observed in the N 1s spectrum (Figure S14). We would stress that the observed signal loss is mostly related to leached guest moieties which are bound to the surface, and that are susceptible to release during a local acidity increase associated with the H_2_O_2_ evolution process. When MA^+^‐PHI is processed in aqueous solutions of pH between 3.5–4, a complete release of these guest moieties is observed, therefore yielding H‐PHI. Moreover, further indication of an incomplete removal MA^+^ moieties upon catalytic reaction may arise upon interpretation of the recorded O1s spectra of both fresh and cycled sample. As seen in Figure S14c, a decrease in C‐O signal at 532.8 eV is apparent, which would otherwise coincide with the simultaneously observed change in the C1s survey (C─NH*_x_* signal) due to degrees of overlap. Thus, catalytic processing does not lead to a full release of MA^+^ species, and the PHI network also undergoes removal of surface bound oxygen species. The powder XRD patterns of the fresh and cycled samples provide similar evidence: the in‐plane crystallinity decreases slightly after cycling, as indicated by the reduced intensity of the (100) and (110) reflections (Figure S15). FT‐IR spectra of the two samples show absorption bands at the same positions, confirming that all functional groups are retained (Figure S16).

**FIGURE 4:**
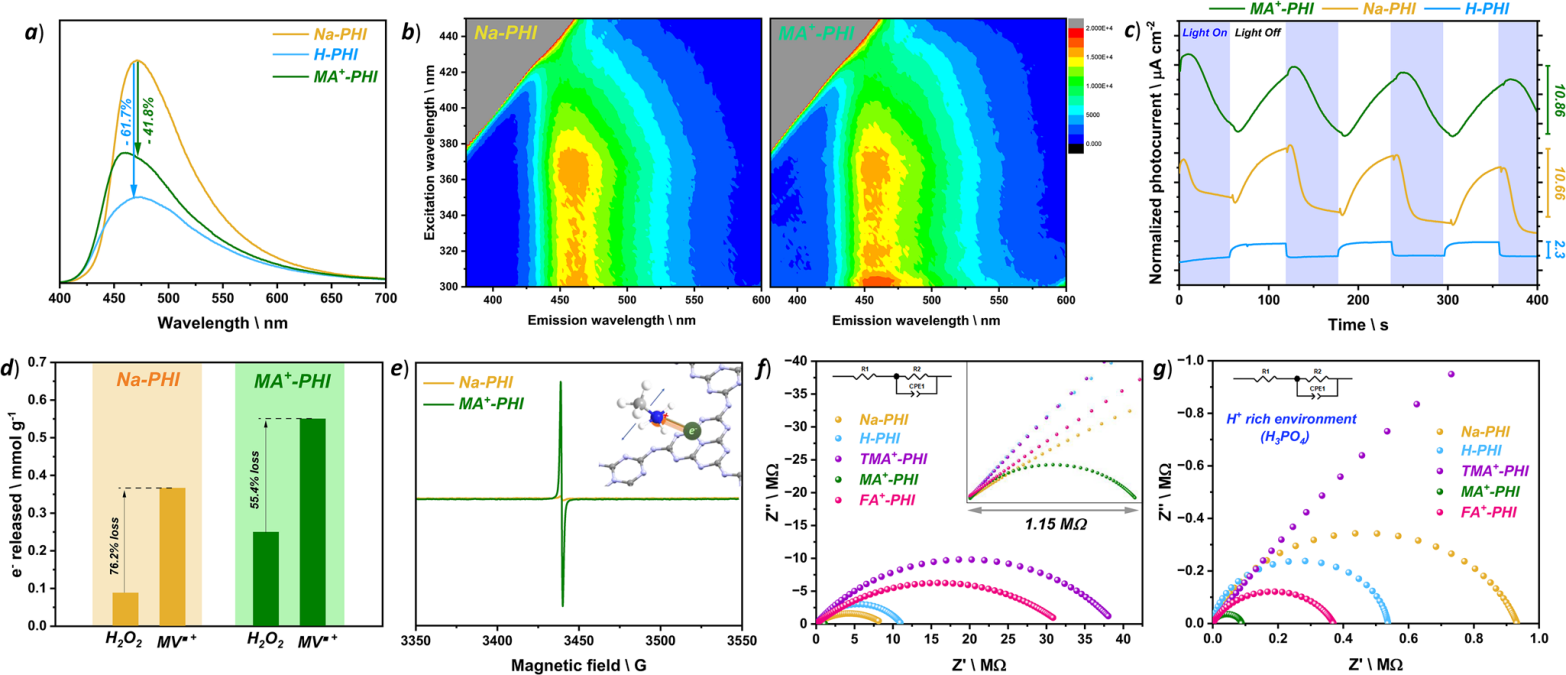
Photoluminescence spectra acquired for Na‐PHI (yellow line), H‐PHI (blue line) and MA^+^‐PHI (green line) with an excitation wavelength of 390 nm (a); 3D photoluminescence plots of Na‐PHI (left) and MA^+^‐PHI (right), with the x‐axis corresponding to emission spectra while the y‐axis corresponds to excitation spectra (b); transient photocurrent plots acquired for Na‐PHI (yellow line), H‐PHI (blue line) and MA^+^‐PHI (green line). Electrodes were prepared on FTO substrates with Nafion as binder, while a bias of ‐0.4 V (vs. Ag/AgCl) was applied for all measurements. A 0.1 M Na_2_SO_4_ solution was used as electrolyte. Incident light was provided through a 427 nm LED. Light on/off cycles are indicated in the plot (c); dark‐photocatalysis experiments carried out after one hour of photo‐charging in a 10% glycerol solution through incident irradiation from a 427 nm LED. Stored electrons were quenched via either O_2_ or methyl viologen (d); EPR spectra of solid Na‐PHI (yellow line) or MA^+^‐PHI (green line) with in‐situ irradiation from a 420 nm light source (e); ionic conductivity (f) and proton conductivity (g) measurements acquired for Na‐PHI (yellow balls), H‐PHI (blue balls), TMA^+^‐PHI (purple balls), MA^+^‐PHI (green balls) and FA^+^‐PHI (pink balls). Impedance analysis was done through a 4‐probe electrode set‐up at open circuit voltage and frequencies between 0.1 Hz and 100 kHz with samples processed into thin pellets without additional binder

To gain further insight into the photo‐physical behavior of investigated PHI photocatalysts, steady state PL measurements were carried out (Figure 4a). After exciting each sample at a 390 nm wavelength, a broad emission signal was recorded for Na‐PHI and H‐PHI at 470 nm, while the maxima of MA^+^‐PHI blueshifted slightly at 458 nm. By integrating areas below recorded curves, increased quenching efficiencies of 41.8% and 61.7% could be attributed to MA^+^‐PHI and H‐PHI, respectively, relative to their Na analogue. Thus, despite H‐PHI exhibiting a smaller emission signal, cation reincorporation as methylammonium moieties would still contribute to a greater charge separation yield than that of Na‐PHI. Moreover, temporal emission decay profiles were also measured for the same samples (Figure S17), which were thereafter fitted to a biexponential function, as described in the Supporting Information. Thus, two lifetime components τ_1_ and τ_2_ could be derived (Table S9). H‐PHI exhibits the longest lifetime for both components, with 5.63 and 1.41 ns determined for τ_1_ and τ_2_, respectively. In conjunction with previously described steady state measurements, it is proposed that despite exhibiting a lower quantum of recombination events, emission arising from photoexcited H‐PHI is longer lived, thus reducing sites available for electron transfer during photocatalysis which could also be observed in Figure 3a. In the case of Na‐PHI and MA^+^‐PHI, τ_1_ shows similar lifetimes, while significant differences were recorded for τ_2_ (0.57 and 0.03 ns). Thus, average emission lifetime of MA^+^‐PHI is significantly lower, in accordance with recorded H_2_O_2_ photocatalytic outputs. 3D photoluminescence plots were also recorded for Na‐PHI and MA^+^‐PHI (Figure 4b). Excitation versus emission overlays point out that both samples have a broad emission signal with maxima at 460 nm. Na‐PHI exhibits two excitation modes with peaks at 305 and 365 nm, associated with π–π* electronic transitions in the heptazine‐based polymer [37]. Additionally, MA^+^‐PHI exhibits another excitation mode at 320 nm which may arise from the stabilization of a transient state via methylammonium conformational flexibility [38].

Transient photocurrent measurements, in accordance with UV–vis spectra discussed in Figure 1d, show a limited photo‐response in the case of H‐PHI, under illumination from a 427 nm light source, equivalent to 2.3 µA cm^−2^. Na‐PHI and MA^+^‐PHI show similar photocurrent intensities, with 10.66 and 10.86 µA cm^−2^, respectively (Figure 4c). To be noted that recorded amperometric profiles of the latter two exhibit slow charging‐discharging profiles when light is either applied or removed. Thus, the quantum of photogenerated electrons under these cathodic conditions is available for photocatalytic turnover over a longer period. Additionally, photo‐charging experiments were done in a 10% glycerin solution under inert atmosphere (Figure 4d). Photo‐charged PHIs exhibit a deep blue color, being an indication of e^−^ stabilization into the heptazine structure [39]. After one hour of continuous irradiation, the e^−^‐rich states of Na‐PHI and MA^+^‐PHI were quenched with either O_2_ or methyl viologen. H_2_O_2_ yields indicate equivalent to 0.08 and 0.25 mmol g^−^^1^ of electron stored and utilized by Na‐PHI and MA^+^‐PHI, while methyl viologen quenching indicated a greater amount of e^−^ charged, with 0.36 mmol g^−^^1^ in the case of Na‐PHI and 0.55 mmol g^−^^1^ for MA^+^‐PHI. During electron transfer towards the O_2_ substrate, losses in recorded charges could arise from either reductive activation of H_2_O_2_, or further e^−^ transfer toward a 4e^−^ balance. Thus, a lower charge loss in the case of MA^+^‐PHI (55.4%) compared to Na‐PHI (76.2%) may be derived from a faster formation and release of product, possible indication of improved PCET in the presence of network stabilized methylammonium moieties. Such an observation is in good accordance with the kinetic model previously described in Figure 3f. EPR spectra recorded for all solids under investigation (Figure S18) show a greater quantum of unpaired e^−^‐spins for the irradiated MA^+^ specimen, compared to all other related analogues. Notably, EPR intensity of MA^+^‐PHI is 48 times higher than that of Na‐PHI (Figure 4e). Conjoint data from photo‐charging and EPR experiments indicate towards long‐lived photogenerated e^−^ stabilized through the conformational flexibility of methylammonium, a phenomenology previously described in solar transducers based on hybrid perovskites [16].

Ionic conductivity measurements were carried out via a four‐probe electrode set‐up, as described in the supportung information. Prior to impedance analysis, investigated specimens were processed into thin pellets. A constant amount of moisture was applied to each sample prior to analysis, as water molecules introduce additional motional/conformational degrees of freedom which favor a clearer dielectric screening of embedded cations and their mobility in the PHI network [19] Nyquist plots of all PHI photocatalysts exhibit a semicircle at high frequencies, which is attributed to bulk transport processes (Figure 4f). Obtained conductivity values are listed in Table S10. MA^+^‐PHI exhibits an ion conduction value roughly one order of magnitude higher than all other PHI analogues under study. Such an observation is in good accordance with in situ EPR measurements, where it is shown that MA^+^ stabilizes photogenerated electrons, assumed by its conformational flexibility. Notably, PHI specimens show a trend following the hydrated ionic radii of each moiety, with 4.4 mS m^−1^ for MA^+^ (r_hcat_≈3.4 Å [40] 290 µS m^−1^ for Na‐PHI (r_hcat_≈3.6 Å [41] 170 µS m^−1^ for FA^+^ (r_hcat_≈3.6 Å) and 93 µS m^−1^ for TMA^+^ (r_hcat_≈3.7 Å [42]). Conductivity in H‐PHI could arise from either trace amounts of Na^+^ as a result of incomplete desodiation or H_3_O^+^ species from its inherent dissociation. As photocatalytic H_2_O_2_ evolution is a process that greatly benefits from efficient PCET mechanistic steps, the dynamic behavior between PHI samples and protons was also assessed. Thus, by premixing the aforementioned pelleted samples with 0.1 mM solution of H_3_PO_4_ of 15% powder weight, proton conductivity values were recorded and listed in Table S10. As observed in Figure 4g, Nyquist plots show the same behavioral shape, yet the trend between analyzed PHIs changes drastically. As previously observed, MA^+^‐PHI showed the best performance (56.8 mS m^−1^), while the H^+^ conductivity of FA^+^‐PHI and H‐PHI is 14.4 and 7.46 mS m^−1^, respectively. This interfacial H^+^ mobility is therefore attributed to structurally liable protons that promote transport via H^+^‐hopping. As Na‐PHI and TMA^+^‐PHI do not show any structural characteristics to support proton‐relay, recorded conductivities proved low. In view of these results, it can be assumed that another factor responsible for the enhanced photocatalytic activity of MA^+^‐PHI is derived from methyl ammonium moieties which act as H^+^‐relay units, therefore facilitating PCET.

**FIGURE 5:**
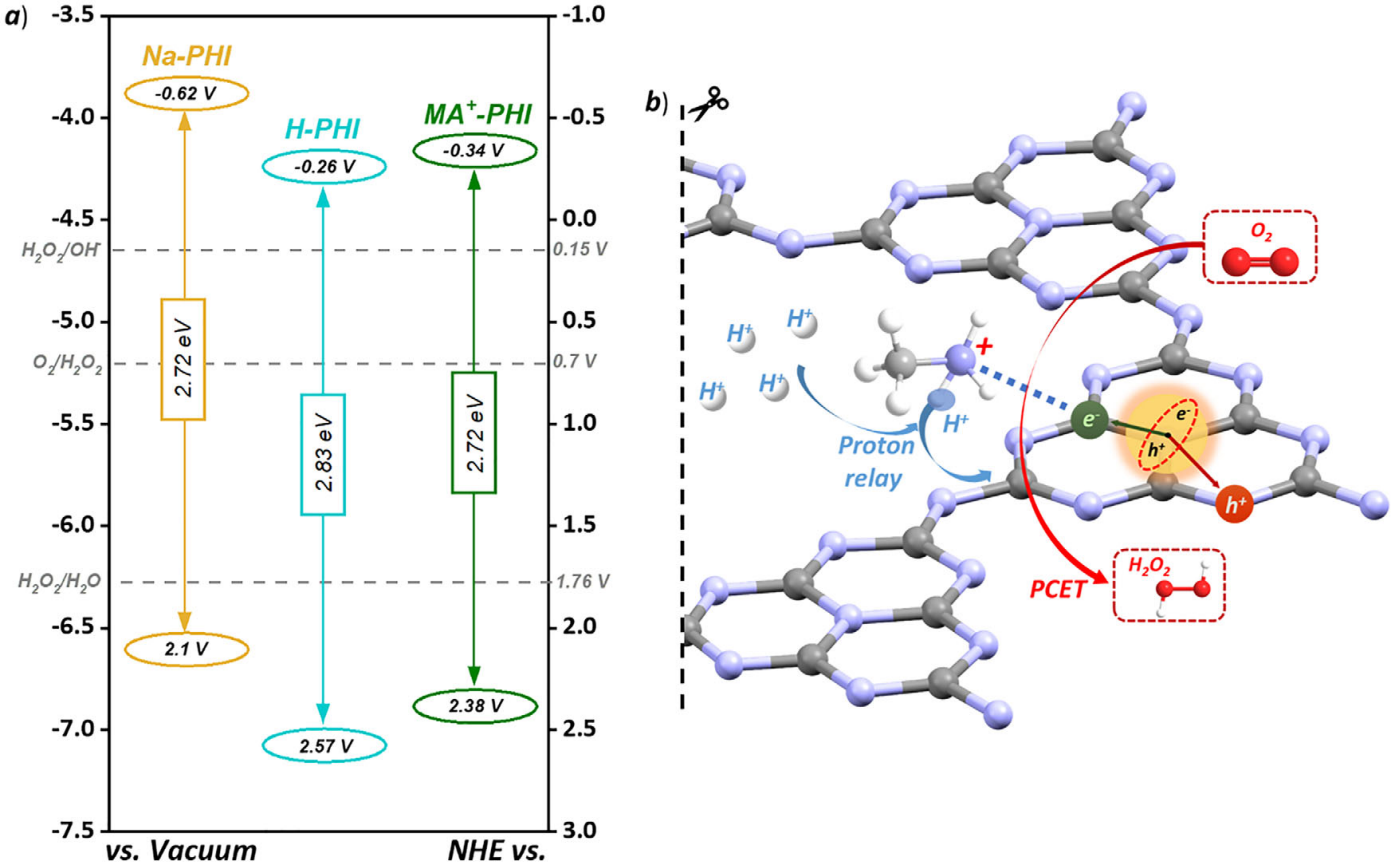
Band energy diagram illustrating the frontier energy values of Na‐PHI, H‐PHI and MA^+^‐PHI. VB positions were calculated from acquired XPS surveys, while CB was determined from the difference between VB and optical band gap (a); schematic structural illustration of MA^+^‐PHI and proposed photocatalytic mechanism (b)

The band diagrams of Na‐PHI, H‐PHI and MA^+^‐PHI were calculated experimentally by determining the optical bandgaps of each material via the Tauc method (Figure S19), while the valence band position was assessed from XPS measurements (Figure S20), the difference between the two corresponding to the conduction band position. As seen in Figure 5a, upon desodiation of Na‐PHI, the heptazine based polymer undergoes changes in electronic properties, and H‐PHI becomes a considerably more oxidative photocatalyst. Upon reincorporation of cations as form of MA^+^‐PHI, both oxidation and reduction potentials shift for values closer to initial Na‐PHI. Considering the overpotentials required for O_2_ reduction, it can be stated that all considered PHI specimens thermodynamically allow this reaction. The greater oxidation potential of MA^+^‐PHI (2.38 V vs. NHE, pH = 0 [43]) than that of Na‐PHI (2.1 V vs. NHE, pH = 0) are in good accordance with the kinetic model previously described in Figure 3f, as the methylammonium containing polymer showed a greater H_2_O_2_ decomposition rate, with a k_d_ of 0.84 h^−^^1^ as compared to Na‐PHI (0.61 h^−^^1^).

In view of photochemical, EPR and H^+^‐conductivity experiments carried out, a plausible mechanism is depicted in Figure 5b. Upon photoexcitation of the PHI semiconductor with spectral contributions not larger than 475 nm (visible light response from UV‐Vis DR and photoaction spectra), the generated excitons undergo separation, leading to charge carriers (e^−^ and h^+^) structurally localized onto heptazine‐units. While h^+^ are quickly scavenged via glycerol oxidation (0.1–0.4 V vs NHE, pH = 0 [43]), excess e^−^ are stabilized by MA^+^ moieties via generation of H_3_C‐H_3_N^+^···e^−^ coulombic pairs, which explain enhanced EPR signals and photo‐charging rates experimentally observed. These stabilized charge carriers with a redox potential of −0.34 V (vs. NHE, pH = 0) can be thereafter transferred to O_2_ species, together with structural H^+^ arising from methylammonium units, facilitating PCET steps for H_2_O_2_ generation. MA^+^ would thereafter recover a H^+^ from the chemical environment, originating from either glycerol or water, thus closing a H^+^‐relay cycle. The Bader charge analysis in Tables S4 and S5 indicates that charge enrichment depends on the proton position but it is largely configuration‐independent and distributed over several framework nitrogen sites. Recorded values for all structural N‐atom types of the PHI network indicate a similar adsorption/activation landscape for substrate O_2_ among all potential interaction sites, while recorded spin‐trap experiments described in Figure S12 clearly highlight the formation of ─^•^OOH adducts, a consequence of end‐on adsorption modes via the central‐N of a heptazine unit. Adjacent pyridinic sites would otherwise favor a side‐on configuration, generally favoring the 4e^−^ ORR pathway, and being detrimental to H_2_O_2_ accumulation in the system [44].

## 3 Conclusions

Inspired by the photochemical complexity of solar transducers based on hybrid perovskites, organic cations such as methylammonium, formamidinium, and tetramethylammonium were successfully incorporated into PHI networks, leading to enhanced photochemical and photocatalytic properties. H_2_O_2_ evolution was studied as a model reaction. MA^+^‐PHI exhibiting the best performance, 7.6 mmol g^−1^ accumulated H_2_O_2_, equivalent to a 42% enhancement compared to the parent Na‐PHI, and an AQY of 11.5% under a visible irradiation contribution of 427 nm. Moreover, upon improving O_2_ availability via pressurizing the batch system to 3 bars, a H_2_O_2_ yield of 36.5 mmol g^−^^1^ (AQY ∼66.8%) was obtained, recommending our synthetic approach as one of the most successful to date for the studied model photocatalytic reaction. While small degrees of cation leaching could be observed after the first catalytic cycle, further operational runs did not show any degree of molecular release, H_2_O_2_ output remaining consistent. Emission spectroscopy, in‐situ EPR measurements and photo‐charging experiments indicate towards a greater quantum of photogenerated e^−^ available for transfer towards O_2_, property arising from the conformational flexibility of methylammonium which stabilizes photoelectrons via coulombic‐pair formation. Moreover, proton conductivity measurements and catalytic experiments point out that MA^+^ moieties act as proton relay units, thus improving H_2_O_2_ generation via PCET steps.

Considering current findings, embedding organic counterions in PHIs is a strategy (known from hybrid perovskites) with further potential ahead. The complex charge carrier dynamics arising from organic cation species need to be deeper understood, while more complex photocatalytic models dependent on PCET mechanistic steps can be studied and optimized, in the view of achieving high‐performing metal‐free photocatalytic technologies.

## Author Contributions

**DIngqiao Ji**: investigation, data curation, writing – orginal draft, writing – review and editing, methodology, formal analysis. **Hikmat Binyaminov**: investigation, writing – review and editing, methodology. **Desiree Leistenschneider**: investigation. **Martin Oschatz**: investigation. **Paolo Giusto**: investigation. **Markus Antonietti**: writing – review and editing, resorces, funding acquisition, supervision. **Horațiu Szalad**: conceptualization, writing – original draft, writing – review and editing, validation, supervision.

## Conflicts of Interest

The authors declare no conflicts of interest.

## Supporting information




**Supporting File**: anie73220‐sup‐0001‐SuppMat.docx.

## Data Availability

The data that support the findings of this study are available from the corresponding author upon reasonable request.
