# Supplementary material for: Incorporation of Organic Counter‐Cations Into Poly(Heptazine Imide) Networks for Promoting Proton‐Coupled Electron Transfer During Photocatalytic H2O2 Evolution
Source: Angew Chem Int Ed Engl. 2026 Jun 13;65(34):e24638. doi: 10.1002/anie.202524638 (PMC13480526; doi:10.1002/anie.202524638)
Supplement: Supplementary file 1 [file ANIE-65-e24638-s001.docx]

***Supporting Information***

**Incorporation of Organic Counter-cations into Poly(heptazine imide) Networks for Promoting Proton-coupled Electron Transfer During Photocatalytic H_2_O_2_ Evolution**

*Dingqiao Ji,^a^* *Hikmat Binyaminov,^a,b^ Desiree Leistenschneider^c,d^ Martin Oschatz,^c,d,e^ Paolo Giusto,^a^ Markus Antonietti^a^ and Horațiu Szalad*^a^*

^a^Department of Colloid Chemistry, Max Planck Institute of Colloids and Interfaces, Research Campus Golm, 14476 Potsdam, Germany.

^b^Theoretical Bio- and Soft Matter Physics, Freie Universitat Berlin, Arnimallee 14, Berlin 14195, Germany.

^c^Institute for Technical and Environmental Chemistry, Friedrich Schiller University Jena, Philosophenweg 7a, 07743 Jena, Germany.

^d^Center for Energy and Environmental Chemistry Jena (CEEC Jena), Friedrich Schiller University Jena, Philosophenweg 7a, 07743 Jena, Germany

^e^Helmholtz Institute for Polymers in Energy Applications Jena (HIPOLE Jena), Lessingstraße 12–14, 07743 Jena, Germany.

*** Corresponding author:**

*Horațiu Szalad:* [Horatiu.szalad@mpikg.mpg.de](mailto:Horatiu.szalad@mpikg.mpg.de)

**Table of Content**

| S.1. General experimental methodology | ***s-3*** |
| --- | --- |
| S.1.1. Synthesis of photocatalysts | ***s-3*** |
| S.1.2. Chemical, structural, and morphological characterization | ***s-3*** |
| S.1.3. Photocatalytic tests | ***s-4*** |
| S.1.4. Photoelectrochemical measurements methodology | ***s-4*** |
| S.2. Chemical composition of studied materials | ***s-5*** |
| S.3. Onium cation determination via NMR aliquots | ***s-6*** |
| S.4. TG-MS analysis recorded for TMA^+^-PHI and FA^+^-PHI | ***s-7*** |
| S.5. Simulated PXRD diffraction patterns | ***s-8*** |
| S.6. SEM images of investigated samples | ***s-9*** |
| S.7. XPS O 1s and Na 1s spectra recorded for Na-PHI, H-PHI and MA^+^-PHI | ***s-9*** |
| S.8. Density functional theory calculations for MA-H-PHI system | ***s-10*** |
| S.9. Methodology for fitting semiconductor absorption to LED light contribution | ***s-15*** |
| S.10. Methodology for AQY% calculation | ***s-16*** |
| S.11. Glycerol oxidation | ***s-18*** |
| S.12. In-situ EPR experiments employing DMPO as a spin trap | ***s-19*** |
| S.13. H_2_O_2_ yield comparison with recently reported carbon nitride catalysts | ***s-19*** |
| S.14. Recycling experiments carried out for the MA^+^-PHI photocatalyst | ***s-22*** |
| S.15. XPS spectra, XRD and FT-IR for MA^+^-PHI before and after catalytic cycling | ***s-23*** |
| S.16. TCSPC methodology and data recorded for samples under study | ***s-25*** |
| S.17. In-situ EPR experiments carried out for solid samples | ***s-26*** |
| S.18. Methodology and procedures for ionic conductivity measurements and electrochemistry | ***s-26*** |
| S.19. Determination of VB and CB through VB-XPS and the Tauc method | ***s-27*** |
| References | ***s-27*** |

**S.1. General experimental methodology**

Chemicals employed in the current study were purchased from commercial providers such as Sigma-Aldrich or TCI (Tokyo Chemical Industry), and were used as received, without any further chemical treatment or purification. Sodium chloride (NaCl, CS reagent, ≥99.0 %), melamine (99 %), concentrated hydrochloric acid (HCl, fuming 37 %), 1,8-Diazabicyclo[5.4.0]undec-7-ene (DBU, puriss., ≥99.0% GC), tetramethylammonium chloride (TMA, reagent grade, ≥98%), methylammonium chloride (MA, for synthesis), formamidinium iodide (FA, ≥99%, anhydrous) and glycerol (≥99.5%) are all the chemicals employed in the experimental investigation. Ultrapure water was collected via a Milli-Q® IQ 7000 Ultrapure Water System. Pure gases such as Ar and O2 were collected from pressurized gas bottles supplied by Linde plc.

**S.1.1. Synthesis of photocatalysts**

***Na-PHI:*** This standard PHI material was synthesized in accordance with previously reported experimental procedures ^[1]^. In a typical manner, 40 g of NaCl and 4 g of melamine were weighed and transferred to an agate mortar. The mixture was manually ground for 10 min to ensure homogeneity, and was then loaded into a ceramic crucible and placed in a Nabertherm N7/H/B500 furnace under a constant N₂ flow of 5L·min⁻¹. The temperature was ramped at 4°C·min^-1^ to 550°C and held for 8 h. After naturally cooling to room temperature under nitrogen, the solid cake was carefully ground into a fine yellow powder, and thoroughly washed with hot deionized water. The recovered yellow solid was thereafter dried at 60°C under vacuum overnight.

***H-PHI:*** To protonate the sodium analogue, 200 mg of Na-PHI was dispersed in 300 mL of 1 M HCl solution under stirring for 12 h at room temperature. The suspension was centrifuged at 9000 rpm for 10 min, and the supernatant was discarded. The solid was washed five times with deionized water until a neutral pH was recorded. The recovered pale solid was recovered and dried at 60°C under vacuum overnight ^[2]^.

***(TMA^+^/Ma^+^/FA^+^)-PHI:*** 30 mg of H-PHI was dispersed in 20mL of deionized water in a round-bottom flask. Afterwards, 100 µL of DBU was added dropwise to facilitate deprotonation. The resulting mixture was stirred for another 10 minutes, after which 2 mmol of the respective onium cation salt (TMA^+^Cl, MA^+^Cl, or FA^+^I) was introduced, and the mixture was stirred until complete dissolution was achieved. After 30 minutes of continuous stirring, the yellow solid was recovered via centrifugation, washed several times with ultrapure water, and dried overnight at 60°C under vacuum.

**S.1.2. Chemical, structural, and morphological characterization**

X-ray powder diffraction (XRD) was performed using a Rigaku SmartLab (Japan, Cu K, 0.154nm) instrument, at an operational voltage of 40 kV and a 50 mA current. Fourier transform infrared (FT-IR) spectra were recorded through a Thermo Scientific NicoletiD5 spectrometer operating on the attenuated total reflection sampling technique. Manual background subtraction was applied to all the curves. Scanning electron microscopy (SEM) images were obtained on a LEO 1550-Gemini microscope. Transmission electron microscopy (TEM) images were taken using a JEOL JEM F200 instrument. UV–VIS DR spectra were acquired via a UV-2600 setup acquired from Shimadzu. Thermogravimetric analysis coupled with a mass spectroscopy attachment (TGA-MS) was carried out via a NETZSCH TG-209 Libra apparatus under He atmosphere, the applied heating ramp for each sample being 10°C·min^-1^. Elemental analysis measurements (EA) were carried out with an Elementar Vario EL III instrument. Inductively coupled plasma optical emission spectrometry (ICP-OES) measurements were recorded via a PerkinElmer ICP-OES Optima 8000 setup. Electron paramagnetic resonance spectra (EPR) were recorded via a Bruker EMX CW-Micro X band EPR spectrometer. Photoluminescence spectra (PL) were acquired by use of a Jasco FP-8300 fluorescence spectrometer, by employing an excitation mode at 390 nm. Time-correlated single photon counting (TCSPC) measurements were done via a FluoTime 250 spectrometer equipped a with PDL 800-D picosecond pulsed diode laser drive. X-ray photoelectron spectroscopy (XPS) data were acquired by employ of a spectrometer setup from Thermo Scientific, with Al Kα as the X-ray source operated at 1486.6 eV. All measurements were carried out under reduced pressure. A flood gun (NEK150-SC) was employed for charge compensation. Liquid phase NMR results were acquired with BBFO 400 MHz-probe.

**S.1.3. Photocatalytic tests**

Standard photocatalytic screening was done in a tube batch-type reactor from Ace Glass, equipped with a pressure valve. The preparation of reaction solutions was made by thoroughly dispersing 2 mg of target photocatalyst in 2 mL of a 10 v.% glycerol aqueous solution. The mixture was thereafter sonicated in a water-bath system for 20 minutes, after which the reactor was flushed several times with O_2_ gas, and an overpressure of 1.5 to 3 bars of the same gas was applied depending on requirement. Photocatalytic experiments were carried out at room temperature, with incident monochromatic irradiation facilitated by Kessil LED light systems. Spectral contributions employed are mentioned for each individual case. The quantification of photo-catalytically evolved H_2_O_2_ was done via a colorimetric method, using an acidified titanium oxide-oxalate solution (TiOSO4). The calibration was carried out through H_2_O_2_ aliquots of known concentrations, UV-Vis absorption values which were then linearly fitted. The obtained curve equation was used in quantifying experimental yields.

**S.1.4. Photoelectrochemical measurements methodology**

**Working electrode preparation (PHI on FTO)**

A catalyst ink was prepared by mixing 2 mg of PHI powder with 25 µL of Nafion (5 wt% solution) and 175 µL of deionized water. The mixture was sonicated for 15 min to obtain a homogeneous slurry. FTO substrates were cleaned by sonication in acetonitrile, dried, and placed on a pre-heated hotplate at 80 °C. The ink was deposited onto the conductive side of the FTO by drop-casting 10 µL per aliquot. Each aliquot was allowed to dry completely before the next deposition. This procedure was repeated five times. The resulting films were further dried at 70 °C for 1 h, yielding a uniform, non-transparent coating.

**Electrochemical setup**

All electrochemical and photoelectrochemical measurements were performed using a Gamry Interface 5000E potentiostat in a three-electrode configuration. The PHI-coated FTO fixed to platinum current collector as the working electrode, Ag/AgCl was used as the reference electrode, graphite rod as counter electrode. Electrolyte was 0.1 M Na2SO4 (aqueous). All measurements were carried out in air at room temperature.

**Cyclic voltammetry (CV)**

CV was performed for 5 cycles at a scan rate of 50 mV s^−1^ over a potential window from −1.5 V to +1.0 V versus Ag/AgCl. The purpose of CV test is to stabilize the electrode/electrolyte interface and remove loosely bound surface species.

**Photocurrent measurements (chronoamperometry under chopped light)**

Photocurrent responses were measured under constant applied potential at −0.3 V versus Ag/AgCl s (non-faradaic region) for a total duration of 660s. The current was allowed to stabilize for 60 s in the dark before recording. Illumination was then alternated in a chopped mode with 60 s light / 60 s dark intervals for the remainder of the experiment.

Illumination was provided by Kessil PR160L (Gen 2) LEDs with selectable wavelengths (400, 427, 456, or 516 nm). The LED was positioned 5 cm from the working electrode. Each experiment was repeated using three independently prepared electrodes; all three electrodes showed the same trend, and the first measurement is shown as the representative dataset after subtracting dark current.

**S.2. Chemical composition of studied materials**

The chemical composition of investigated samples was determined via both EA and ICP-OES. Indirect quantification of embedded onium ions was determined through variations in the C:N ratio compared to the recorded values of Na-PHI and H-PHI standards.

**Table S1.** Analytical data recorded for materials under study.

| **Sample** | **N content (wt.%)** | **C content (wt.%)** | **N:C molar ration** | **Cation^+^/unit cell** | **Cation content (wt.%)** |
| --- | --- | --- | --- | --- | --- |
| ***Na-PHI*** | 44.3 | 27 | 1.405 | 2.06 | 11 |
| ***H-PHI*** | 48.8 | 29.7 | 1.41 | None | None |
| ***DBU-H-PHI*** | 51.1 | 32.4 | 1.35 | 0.078 (*surface impurities*) | 3 (*surface impurities*) |
| ***TMA^+^-PHI*** | 46.3 | 34 | 1.17 | 0.6 | 8.5 |
| ***MA^+^-PHI*** | 50.5 | 32.7 | 1.32 | 0.9 | 7 |
| ***FA^+^-PHI*** | 50.3 | 31.1 | 1.39 | 1 | 9.7 |

**S.3. Onium cation determination via NMR aliquots**

In a similar procedure as discussed in section **S.1.1.**, the preparation of TMA^+^-PHI, Ma^+^-PHI and FA^+^-PHI were carried out in D_2_O, whereas aliquots were recovered before and after incorporation of the onium ions into the PHI network. ^1^H NMR spectra were thereafter recorded, compared, and the ammonium cations in solution were quantified by employing acetonitrile as an internal standard.

**Figure S1.** ^1^H-NMR spectra recorded before (dark line) and after the incorporation of TMA^+^ (purple line) (a), MA^+^ (green line) (b) and FA^+^ (pink line) (c).

**Table S2.** Onium ion incorporation calculated from recorded ^1^H NMR spectra.

| **Sample** | **Peak ratio (ACN/Cat^+^)** | **Reacted Cat^+^ (mg)** | **Cat^+^/lattice** | **Cation content (wt.%)** |
| --- | --- | --- | --- | --- |
| ***TMA^+^*** | 0.374 | None | None | None |
| ***TMA^+^ + H-PHI*** | 0.341 | 4.2 | 0.73 | 12.3 |
| ***MA^+^*** | 0.266 | None | None | None |
| ***MA^+^ + H-PHI*** | 0.231 | 3.2 | 1.27 | 9.6 |
| ***FA^+^*** | 0.229 | None | None | None |
| ***FA^+^ + H-PHI*** | 0.206 | 2.7 | 0.94 | 9.7 |

**S.4. TG-MS analysis recorded for TMA^+^-PHI and FA^+^-PHI**

**Figure S2.** TGA decomposition analysis recorded for TMA^+^-PHI (a) and FA^+^-PHI (b). The TG weight-loss curves are illustrated with blacklines, while DTG signals are shown via blue lines. All measurements were recorded in He atmosphere.

**Figure S3.** Mass spectrometer response at different temperatures recorded for TMA^+^-PHI (a) and FA^+^-PHI (b). All mass fractions of interest are illustrated via colored lines.

**S.5. Simulated PXRD diffraction patterns**

The unit cell .cif file was generated from the idealized PHI unit cell with three methylammonium cations via one-click simulation through the software VESTA version: 4.6.0.

**Figure S4.** Simulated powder XRD pattern of MA^+^-PHI (a) and H-PHI (b). Results shows consistency of predicted peak positions.

**S.6. SEM images of investigated samples**

**Figure S5.** fresh Na-PHI (a) showing a flake-like morphology induced by preferential growth onto NaCl crystals during polycondensation of precursors, while processed samples reveal a disordered agglomeration of smaller particles (b-e).

**S.7. XPS O 1s and Na 1s spectra recorded for Na-PHI, H-PHI and MA^+^-PHI**

**Figure S5.** O 1s XPS spectra acquired for Na-PHI (a), H-PHI (b) and MA^+^-PHI (c).

**Figure S6.** Na 1s XPS spectra acquired for the Na-PHI sample.

**S.8. Density functional theory calculations for MA-H-PHI system**

**Methodology:** Electronic structure calculations were performed using the Gaussian and Plane Waves (GPW) formalism as implemented in the CP2K/Quickstep package ^[3]^ ^[4]^ ^[5]^. The Perdew–Burke–Ernzerhof [^6]^ (PBE) exchange-correlation functional was employed within the Generalized Gradient Approximation (GGA). To accurately capture non-covalent interactions, long-range dispersion was accounted for via the DFT-D3 method with Becke–Johnson (BJ) damping ^[7]^ ^[8]^. Valence electrons were expanded in molecularly optimized double-zeta valence polarized (DZVP-MOLOPT-GTH) basis sets ^[9]^ , while core electrons were modeled using norm-conserving Goedecker–Teter–Hutter (GTH) pseudopotentials ^[10]^ ^[11]^.

For production runs, the auxiliary plane-wave grid was defined by a high-density cutoff of 800 Ry and a relative cutoff of 80 Ry. The Brillouin zone was sampled with a 3x3x1 Monkhorst–Pack k-point grid^[12]^, which was found to be converged to within 10^-5^ Ha compared to a 9x9x1 grid. Band structures and densities of states (DOS) were evaluated along the high-symmetry path. Preliminary screening and initial structural relaxations were performed using a reduced cutoff (300/50 Ry) and point-only sampling to accelerate convergence. The Bader charge analysis was performed using the quantum theory of atoms in molecules (QTAIM) as implemented in the CRITIC2 program ^[13]^ ^[14]^. Visualizations were created using a workflow combining VMD ^[15]^ and Blender ^[16]^ with custom scripts.

**Figure S7.** Initial bare PHI structure in the primitive cell with atom indices shown (a), and a portion of a supercell depicting starting positions used in configurational search (b). The carbon and nitrogen atoms are depicted as gray and blue spheres, respectively. The highlighted atoms in dashed circles indicate the selected nitrogen site for the bonding of the hydrogen atom, where applicable.

**The model:** The model system consists of a primitive unit of a single-layer PHI crystal with one hydrogen atom and two methylammonium (MA^+^) groups added (total 14 carbon, 19 nitrogen, 13 hydrogen atoms) in periodic boundary conditions. Calculations were performed using a unit cell with a hexagonal lattice metric in the ab plane with fixed lattice constants a=b=12.57 Å. These lattice parameters are taken from the Na-PHI system ^[17]^ and cell optimization shows only a slight deviation (~ 0.1%) for the current system owing to the rigidity of the PHI framework. A large out of plane lattice parameter of c=20 Å was employed to minimize interlayer interactions resulting in a quasi-two-dimensional periodic model. The initial bare PHI structure consisting of 12 carbon and 17 nitrogen atoms within the unit cell is shown in **Figure S7.a**. Atoms are indexed for reference in **Table S7** and these indices are kept throughout the study.

**Configurational search:** Unlike single atom metal cations, the (co)adsorption sites and (co)orientations of two multiatom organic MA^+^ groups within the PHI unit cell are not trivial, requiring a systematic study. Additionally, it is not known which framework nitrogen the lone proton is covalently bonded to. To that end, we checked two plausible protonation sites, namely the bridge nitrogen connecting two heptazine units (position F in **Figure S7.b**), and the opposing site between two nitrogens belonging to neighboring heptazine units (position A in **Figure S7.b**).

For preliminary screening of possible MA^+^ adsorption sites and orientations, we started with many randomly generated configurations where we placed in random positions and orientations the two MA^+^ groups within 5 Å of the framework (distance in the normal direction to the PHI plane) with the proton in one of the positions noted above (A or F). We then geometry-optimized these configurations to bring them to local minima. We observed in the resulting geometries that, in all cases, each MA^+^ is adsorbed by reorienting its NH_3_ group towards the framework in approximately one of the positions shown in **Figure S7.b**. The two CH_3_ groups of the cations always point out of the framework plane creating a cis or trans configuration. Based on these initial findings, we generated all possible (16 total) unique adsorption configurations covering both protonation states and all cis-trans isomers by placing the nitrogens of the two MA^+^ groups in the positions shown in **Figure S7.b** with their C–N bond perpendicular to the framework plane. We deliberately did not include configurations where the two MA^+^ groups would have been in close proximity of each other (e.g., one of them in position B and the other one in position D) to prevent their direct chemical interaction. The summary of the tested configurations is given in **Table S3** (the positions in the table refer to **Figure S7.b**).

We optimized all 16 configurations listed in **Table S3** and picked the two lowest-energy geometries for each protonation state discarding the remaining configurations for simplicity. These four configurations turned out to be the ones listed in the second column of **Table S3**, namely A-BC-cis, A-BC-trans, F-AC-cis, and F-AC-trans configurations. The optimized systems were then thermalized at 500 K followed by dynamical temperature annealing for 20 ps using the second-generation Car–Parrinello molecular dynamics method of Kühne et al. ^[18]^ ^[19]^ to find the nuclear ground state. A final geometry optimization was performed on the annealed structures with tight convergence parameters (maximum force < 4.5×10^-4^ Ha/Bohr and maximum displacement < 3×10^-3^ Bohr).

**Table S3.** Summary of all configurations considered for the configurational search. Refer to **Figure S7.b** for visualization of the positions. The four lowest energy configurations are highlighted in red.

| **H position** | **MA config. 1** | **MA config. 2** | **MA config. 3** | **MA config. 4** |
| --- | --- | --- | --- | --- |
| A | BC-cis | BE-cis | DE-cis | EF-cis |
| A | BC-trans | BE-trans | DE-trans | EF-trans |
| F | AC-cis | BC-cis | CD-cis | DE-cis |
| F | AC-trans | BC-trans | CD-trans | DE-trans |

**Results:** The resulting four configurations are shown in **Figure S8** together with their highest occupied molecular orbital (HOMO) and lowest unoccupied molecular orbital (LUMO) iso-surfaces. The energy difference between cis and trans isomers at protonation site A was found to be 0.8 kJ/mol. This value falls within the expected accuracy limits of the PBE functional, suggesting that these isomers are effectively degenerate. In contrast, at protonation site F, the trans isomer is energetically preferred by 6 kJ/mol. Despite this preference, MD annealing simulations revealed frequent stochastic transitions between cis and trans configurations with proton at either site. These observations suggest that, for such MA-H-PHI monolayers, both isomers are likely to coexist in a thermal equilibrium at room temperature. Furthermore, while the A-BC-trans configuration is 18 kJ/mol more stable than the F-AC-trans configuration, the physical relevance of this global minimum depends on the experimental feasibility of accessing both protonation states under synthesis conditions.

The band structure and the total density of states are plotted in **Figure S9** for all four systems. As is typical for semi-local functionals like PBE, the calculated band gap is expected to be a significant underestimation of the true fundamental gap due to the lack of a derivative discontinuity and the presence of self-interaction error. While the PBE gap of 2.1-2.3 eV shows numerical proximity to the experimental bulk values, this agreement is likely fortuitous, resulting from a cancellation of errors between the PBE underestimation and the quantum confinement effects of the monolayer model, therefore, only qualitative trends are meaningful here.

**Table S4.** Bader charges of four lowest energy configurations in units of |e|. Refer to **Figure S7.a** for atom numbers of the PHI framework. MA^+^ groups and atoms are not labeled in the figures. We also list fully protonated H-PHI charges named 3H-ABC and 3H-DEF for comparison (see the positions in **Figure S7.b**).

| **Group** | **Atom type** | **No.** | **3H-ABC** | **3H-DEF** | **A-BC-cis** | **A-BC-trans** | **F-AC-cis** | **F-AC-trans** |
| --- | --- | --- | --- | --- | --- | --- | --- | --- |
| PHI | C | 1 | 1.291 | 1.268 | 1.257 | 1.252 | 1.293 | 1.276 |
| PHI | C | 2 | 1.271 | 1.262 | 1.243 | 1.252 | 1.254 | 1.24 |
| PHI | C | 3 | 1.271 | 1.29 | 1.263 | 1.27 | 1.283 | 1.252 |
| PHI | C | 4 | 1.237 | 1.298 | 1.248 | 1.262 | 1.252 | 1.275 |
| PHI | C | 5 | 1.232 | 1.269 | 1.247 | 1.248 | 1.241 | 1.237 |
| PHI | C | 6 | 1.253 | 1.282 | 1.246 | 1.251 | 1.229 | 1.24 |
| PHI | C | 7 | 1.24 | 1.284 | 1.249 | 1.264 | 1.243 | 1.286 |
| PHI | C | 8 | 1.262 | 1.28 | 1.253 | 1.26 | 1.246 | 1.257 |
| PHI | C | 9 | 1.231 | 1.277 | 1.279 | 1.25 | 1.253 | 1.259 |
| PHI | C | 10 | 1.28 | 1.282 | 1.298 | 1.281 | 1.263 | 1.267 |
| PHI | C | 11 | 1.271 | 1.274 | 1.25 | 1.243 | 1.239 | 1.246 |
| PHI | C | 12 | 1.283 | 1.292 | 1.277 | 1.27 | 1.259 | 1.233 |
| PHI | Central N | 13 | -0.975 | -0.953 | -0.99 | -0.997 | -1.01 | -0.984 |
| PHI | Ring N | 14 | -0.948 | -0.987 | -0.979 | -0.968 | -1.034 | -1.008 |
| PHI | Ring N | 15 | -0.939 | -0.962 | -1.024 | -1.023 | -1.039 | -1.042 |
| PHI | Ring N | 16 | -1.049 | -0.979 | -1.001 | -1.017 | -1.019 | -1.002 |
| PHI | Ring N | 17 | -0.94 | -0.988 | -1.038 | -1.035 | -0.968 | -0.984 |
| PHI | Ring N | 18 | -1.011 | -0.978 | -1.019 | -1.017 | -1.01 | -1.025 |
| PHI | Ring N | 19 | -1.02 | -0.961 | -1.006 | -1.009 | -0.958 | -0.959 |
| PHI | Central N | 20 | -0.965 | -0.982 | -0.997 | -0.997 | -0.982 | -0.974 |
| PHI | Ring N | 21 | -0.951 | -0.98 | -1.008 | -1.009 | -1.019 | -1.011 |
| PHI | Ring N | 22 | -0.946 | -0.988 | -0.971 | -0.971 | -0.992 | -1.031 |
| PHI | Ring N | 23 | -1.006 | -0.961 | -1.029 | -1.037 | -1.014 | -1.013 |
| PHI | Ring N | 24 | -1.047 | -0.975 | -1.014 | -1.021 | -0.971 | -0.965 |
| PHI | Ring N | 25 | -1.051 | -0.973 | -1.065 | -1.061 | -1.011 | -1.027 |
| PHI | Ring N | 26 | -0.949 | -0.976 | -1.015 | -1.011 | -0.977 | -0.97 |
| PHI | Bridge N | 27 | -0.955 | -1.04 | -1.015 | -1.022 | -1.041 | -1.041 |
| PHI | Bridge N | 28 | -0.971 | -1.005 | -1.018 | -1.021 | -1.053 | -1.064 |
| PHI | Bridge N | 29 | -0.97 | -1.036 | -1.047 | -1.02 | -0.993 | -1.014 |
| H1 | H | — | 0.522 | 0.459 | 0.511 | 0.514 | 0.433 | 0.445 |
| H2 | H | — | 0.519 | 0.448 | — | — | — | — |
| H3 | H | — | 0.527 | 0.458 | — | — | — | — |
| MA1 | C | — | — | — | 0.247 | 0.224 | 0.233 | 0.248 |
| MA1 | N | — | — | — | -1.027 | -1.029 | -1.022 | -1.05 |
| MA1 | Carbon H | — | — | — | 0.05 | 0.083 | 0.052 | 0.062 |
| MA1 | Carbon H | — | — | — | 0.046 | 0.058 | 0.094 | 0.052 |
| MA1 | Carbon H | — | — | — | 0.073 | 0.056 | 0.041 | 0.052 |
| MA1 | Nitrogen H | — | — | — | 0.408 | 0.419 | 0.422 | 0.438 |
| MA1 | Nitrogen H | — | — | — | 0.495 | 0.498 | 0.502 | 0.511 |
| MA1 | Nitrogen H | — | — | — | 0.516 | 0.501 | 0.493 | 0.493 |
| MA2 | C | — | — | — | 0.212 | 0.252 | 0.247 | 0.221 |
| MA2 | N | — | — | — | -0.992 | -1.011 | -1.021 | -1.013 |
| MA2 | Carbon H | — | — | — | 0.082 | 0.047 | 0.068 | 0.062 |
| MA2 | Carbon H | — | — | — | 0.07 | 0.065 | 0.054 | 0.086 |
| MA2 | Carbon H | — | — | — | 0.048 | 0.043 | 0.036 | 0.036 |
| MA2 | Nitrogen H | — | — | — | 0.497 | 0.417 | 0.509 | 0.503 |
| MA2 | Nitrogen H | — | — | — | 0.414 | 0.496 | 0.499 | 0.492 |
| MA2 | Nitrogen H | — | — | — | 0.477 | 0.498 | 0.396 | 0.404 |

**Table S5.** Total Bader charges per group in units of |e|. We also list fully protonated H-PHI charges named 3H-ABC and 3H-DEF for comparison (see the positions in **Figure S7.b**).

| **Group** | **3H-ABC** | **3H-DEF** | **A-BC-cis** | **A-BC-trans** | **F-AC-cis** | **F-AC-trans** |
| --- | --- | --- | --- | --- | --- | --- |
| PHI | -1.571 | -1.366 | -2.126 | -2.133 | -2.036 | -2.046 |
| H1 | 0.522 | 0.459 | 0.511 | 0.514 | 0.433 | 0.445 |
| H2 | 0.519 | 0.448 | — | — | — | — |
| H3 | 0.527 | 0.458 | — | — | — | — |
| MA1 | — | — | 0.808 | 0.81 | 0.815 | 0.806 |
| MA2 | — | — | 0.808 | 0.807 | 0.788 | 0.791 |

**Figure S8.** Four lowest-energy configurations and their HOMO (red) and LUMO (green) iso-surfaces plotted at ±0.06 e/Bohr^3^: A-BC-cis (a), A-BC-trans (b), F-AC-cis (c), F-AC-trans (d). Two views are shown for each configuration, one along the third lattice vector (top) and the other one along the second lattice vector (side).

**Figure S9.** Band structure and density of states (DOS) of four lowest energy configurations shifted by the valence band maximum (VBM) calculated at the PBE level: A-BC-cis (a), A-BC-trans (b), F-AC-cis (c), F-AC-trans (d).

**S.9. Methodology for fitting semiconductor absorption to LED light contribution**

In order to properly asses the effect of onium ions towards the photocatalytic properties of prepared PHI materials, the absorption capability of each sample was related to the contribution of incident photons delivered by the employed LED source.

Thus, a sigmoidal function (logistic‐ or Boltzmann‐type) was chosen for highlighting the steep edge absorption contribution of PHI samples. A typical fit is described as:

$$y\left( \lambda\right)=A_{1}+\frac{A_{2}-A_{1}}{1+e^{(\lambda-\lambda_{0})/d}}$$

**(S1)**

Where A_1_ is the lower (right) plateau value, A_2_ is the upper (left) plateau value of the absorbance, λ_0_ is the “center” (wavelength at which the curve drops halfway), d controls the steepness of the drop.

Afterwards, the spread spectral contribution facilitated by the LED source was described as a Gaussian function in terms of wavelengths (λ):

$$L\left( \lambda\right)=Ae^{-\frac{{(\lambda-\lambda_{0})}^{2}}{2\sigma^{2}}}$$

**(S2)**

Where A is the normalization constant, λ_0_ is the wavelength value at the peak location and σ is the standard deviation controlling the spreading.

Considering the recorded DR UV-Vis spectra of synthesized PHI materials, the amount of light absorbed by each sample during irradiation is determined by knowing the quantum of light not transmitted. Thus, the relation between absorbance and transmittance needs to be considered:

$$1-T=1- \frac{{10}^{(2-A)}}{100}$$

**(S3)**

Where A is absorbance and T is the transmittance.

Therefore, a normalization parameter can be recorded considering the UV-Vis absorbance of the considered semiconductor y(t), the light absorbed from a certain light contribution arising from the LED source with L(λ) and a considered range between λ_1_ and λ_2_.

| $\int_{\lambda_{1}}^{\lambda_{2}} y(\lambda)\times L(\lambda)d\lambda$ | **(S4)** |
| --- | --- |

Considering our own experimental case, the previously described parameters were fitted according to DR UV-Vis spectra recorded for Na-PHI, TMA^+^-PHI, MA^+^-PHI, FA^+^-PHI, and the described light contribution of employed Kessil LED source with a contribution centered at 427 nm. The following normalization parameters were therefore obtained and listed in **Table S6**.

**Table S6.** Normalization parameters calculated for PHI photocatalysts taking into consideration individual light absorption and the spectral contribution of the employed 427 nm LED.

| **Sample** | **A_1_** | **A_2_** | **λ_0_** | **d** | **Abs_427nm_** |
| --- | --- | --- | --- | --- | --- |
| ***Na-PHI*** | 0.2 | 1.1 | 448 | 10 | 0.907 |
| ***TMA^+^-PHI*** | 0.17 | 1.2 | 436.8 | 10 | 0.854 |
| ***MA^+^-PHI*** | 0.19 | 1.03 | 445 | 9 | 0.881 |
| ***FA^+^-PHI*** | 0.19 | 1.12 | 438 | 10 | 0.875 |

**S.10. Methodology for AQY% calculation**

Apparent quantum yield (AQY%) measurements were carried out in the same reactor set-up, employing LED lights of different spectral contributions. AQY% values were calculated based on recorded H_2_O_2_ yields, according to the following equations (***S5-S7***):

$$E=\frac{h\cdot c}{\lambda}$$

***(S5)***

$$N_{p}=\frac{P}{E}$$

***(S6)***

$$AQY\left( \% \right)=\frac{N_{e}}{N_{p}}\cdot100$$

***(S7)***

Where E is the energy of one photon (J), c is the speed of light (m·s^-1^), h is the Planck constant (J·s), λ is the wavelength of the incident irradiation (m), Np is the number of incident photons (photons·s^-1^), P is the measured power of the incident light (W), Ne is the number of e^-^ contributing to evolution of products in one second (e^-^·s^-1^).

Employed reaction mixtures employed in AQY assessment followed the same methodology as in the case of conventional photocatalytic tests, in which 2 mg of catalyst were dispersed in an aqueous solution containing 10 v.% glycerol. H_2_O_2_ yields were quantified after one hour of incident light irradiation. The reactor was placed at 19.5 cm distance from the LED irradiation source, resulting in an irradiation area of 6.13 cm^2^, derived from the volumetric level filled inside the reactor (3.5x1.5 cm) as well as the concave shape of the irradiated interface (**Picture S1**). Moreover, the associated light intensities (mW·cm^-2^) corresponding to each spectral contribution studied are listed in **Table S7**.

**Picture S1.** Photograph of the employed reactor during AQY% assessment, highlighting the irradiated surface during photocatalytic testing.

**Table S7.** Light intensities measured for spectral contributions employed during AQY% assessment of studied photocatalysts.

| **Spectral contribution (nm)** | **Light intensity (mW·cm^-2^)** |
| --- | --- |
| ***400*** | 6.43 |
| ***427*** | 6.7 |
| ***456*** | 6.99 |
| ***518*** | 4.43 |

**S.11. Glycerol oxidation**

**Lactic acid HSQC：** (¹H ~1.30–1.38, ¹³C ~20–22)

**Glycolic acid HSQC:** (¹H ~4.10–4.50, ¹³C ~60–65)

**¹³C acquisition window is −7.3 to 157.5 ppm**, any carbonyl **above 157 ppm will be aliased (folded)** back into this window.

δtrue ≈ δobs + 164.8 = 3.2+164.8 = 168 ppm

**Formic acid HSQC:** (¹H ~8.20–8.50, ¹³C ~168–172)

**Figure S10.** ^1^H Liquid NMR of the system after reaction, showing internal standard at 2ppm. Lactic acid, formic acid as the major byproducts.

**Figure S11.** HSQC of the same system, further supporting the assessed oxidation products.

**S.12. In-situ EPR experiments employing DMPO as a spin trap**

All measurements were carried out by following the same experimental procedure. A quantity of 5 mg of each investigated material were thoroughly dispersed in 1 mL of an acetonitrile solution in which 10 mg of DMPO (5,5-dimethyl-1-pyrroline N-oxide) were dissolved prior. Small aliquots of this dispersion were thereafter transferred into glass capillaries, and spectra acquisition was done before and after irradiating the sample with a 415 nm light source for two minutes.

**Figure S12.** EPR spectra recorded for all samples under investigation before irradiation (a), after irradiation (b).

**S.13. H_2_O_2_ yield comparison with recently reported carbon nitride catalysts**

**Table S8.** Comparative report between the currently reported H_2_O_2_ yield achieved through MA+-PHI, and previously reported precedents.

| **Photocatalyst** | **Catalyst Modification** | **Solution Conditions** | **Irradiation Source** | **H₂O₂ Production Rate** | **Reference** |
| --- | --- | --- | --- | --- | --- |
| Organic cation intercalated PHI from NaPHI | Cation replacement from Na to H to Methylammonium | 10%Glycerin water solution saturated O2, 3bar | 427nm LED | 36.5mmol g⁻¹·h⁻¹ | This Work |
| Alkali-halide-modulated PHI | Alkali metal halide (e.g. KCl) incorporated during thermal polycondensation of melon | 10 M ethanol in water as electron/proton donor (hole scavenger); O₂-saturated solution. | Simulated solar (UV–visible) and visible (λ ≥ 400 nm) light. | 73.4 mM·h⁻¹ | Sharma et al.^[20]^ |
| Hydrophobized PHI (K-PHI/silica composite) | PHI nanoparticles rendered hydrophobic by grafting with alkylated silica | Biphasic: 1-octanol and 0.5–1.0 M HCl; H₂O₂ continuously extracted into water phase. | Visible LED (λ = 406 nm, ~4.2 mW·cm⁻²). | ~0.12 M H₂O₂ | Krivtsov et al.^[21]^ |
| K⁺-doped PHI (K-PHI) | In situ K doping via molten-salt (KCl) synthesis yields ionic PHI framework | Alkaline aqueous solution (pH≳10, e.g. NaOH; no hole scavenger); O₂ from air. | Visible-light irradiation (400 nm < λ < 500 nm). | 6.78 mmol·g⁻¹·h⁻¹ | Fang et al.^[22]^ |
| PHI/PTI isotype heterojunction (C₃N₅) | Molecular-level PHI–PTI junction (“C₃N₅”) . Z-scheme band alignment forms internal E-field (improved charge separation). | Aqueous solution, air atmosphere (ambient O₂); no hole scavengers (neutral pH). | Visible-light illumination (ambient conditions). | 120.4 mM·h⁻¹ | Lv et al.^[23]^ |
| B-doped g-C₃N₄/FeOOH–CoOₓ | B heteroatom doping; FeOOH & CoOₓ cocatalyst clusters | Pure water (pH 7, O₂-saturated) | Visible light (λ ≥ 420 nm, 100 mW cm^−2) | 0.34 mmol·g⁻¹·h⁻¹ (340 μmol·g⁻¹·h⁻¹) | Liang et al. ^[24]^ |
| Alkali-doped g-C₃N₄ (with N vacancies) | K⁺ (metal-free) doping and N-defect engineering | H₂O + 10% isopropanol (electron donor) | Visible light (λ > 420 nm) | 10.2 mmol·g⁻¹·h⁻¹ | Wu et al.^[25]^ |
| Ni single-atom on porous g-C₃N₄ | Ni single atoms (≈12.5 wt%) on ultrathin, porous CN nanosheets | Pure water (O₂-saturated) | AM1.5G sunlight | 640.1 μmol·L⁻¹·h⁻¹ | Zhang et al.^[26]^ |
| Na,S co-doped g-C₃N₄ (CNNS-2.5) | Co-doping with Na and S (via urea + Na₂S₂O₃ one-pot synthesis) | Pure water (e.g. containing organic pollutant in tests) | Visible light (λ > 400 nm) | 24.25 mmol·g⁻¹·h⁻¹ | Chen et al.^[27]^ |
| 0.3Ad/KPHI (carbon/KPHI heterojunction) | Adenine-derived porous carbon integrated with KPHI (heterojunction) | 2 mL 3.5 wt% glycerin, O2 bubbling | 410 nm violet LED | 3.94 mmol·g⁻¹·h⁻¹ | Pelicano et. al.^[28]^ |
| H-Rho-CN-0.5 (PHI-PTI/LiCl system) | Protonation of Rho-CN-0.5 to H-Rho-CN-0.5 (paper naming) | 2 mL H2O:MeOH = 4:1 (v/v) | White LED | 681 µmol g^-1^ h^-1^ | Galushchinskiy et. al.^[29]^ |
| K-PHI/PTI:LiCl heterostructure | Crystalline K-PHI integrated with PTI:LiCl to form heterostructure | 20v% MeOH in water | Visible light (White LED) | 7.8 mol L^-1^ g^-1^ h^-1^ | Szalad et. al.^[30]^ |
| Ox-KPHI (controlled disorder/defects in KPHI) | Controlled lattice disorder/defects to tune KPHI surface reactivity | 10% (w/w) glycerin | 410 nm LED | 10 mM h^-1^ | Tong et al.^[31]^ |
| CN/Zn-OAc | Single Zn atoms on carbon nitride with acetate-anion-enabled asymmetric Zn-N3O coordination. | O_2_-saturated aqueous isopropanol | Visible light | 187.8 μmol g^-1^ min^-1^ | Li et al. ^[32]^ |
| Pyrene-based COF | Pyrene units distributed within the COF framework; biphasic design used to suppress H2O2 decomposition. | Water / benzyl alcohol biphasic system (9:1) | 420 nm visible light | 1574 μmol g^-1^ h^-1^ | Qiao et al. ^[33]^ |
| Porphyrin-based COF membrane | Nanostructured COF membrane with asymmetric wettability for triphase photocatalysis. | Air and water (ambient air) | Natural sunlight (98 mW cm^-2) | 12.5 mmol g^-1^ h^-1^ | Sun et al. ^[34]^ |
| PyIm-COF | Pyridyl-imine structures introduced into a bipyridine-based COF to favor direct one-step 2e- ORR. | Air-equilibrated aqueous system | Xe lamp (>420 nm) | 5850 μmol g^-1^ h^-1^ | Wu et al. ^[35]^ |
| 2.5%AC (K-PHI) | Fragmentation of K-PHI crystals via NH4Cl and introduction of -C≡N. | 10% (w/w) glycerin | 410 nm LED | 12.3 mM | Bhatri et al. ^[36]^ |
| CK–PHI-5 | Plasma induced distortion of K-PHI to corrugate configurations | 10 v% benzyl alcohol in water | >420 nm visible light | 11.6 mmol g^-1^ h^-1^ | Fang et al. ^[37]^ |
| NaK-PHI | Na, K electron sink | Pure water | >420 nm visible light | 672.5 μmol g^-1^ h^-1^ | Fang et al. ^[38]^ |

**S.14. Recycling experiments carried out for the MA+-PHI photocatalyst**

Recyclability tests were carried out by recovering the material employed in the initial photocatalytic run via centrifugation, after which the solid was washed several times with deionized water. Following this, the reaction mixture was prepared and charged into the photoreactor by following the standard routine described in section **S.1.3**. Catalytic cycles were carried out for one hour each.

**Figure S13.** Recyclability tests carried out for the MA^+^-PHI photocatalyst.

**S.15. XPS spectra, XRD and FT-IR for MA^+^-PHI before and after catalytic cycling**

**Figure S14.** Shows a structural conservation of MAPHI. The only change observed is C-NH_x_ bond. In the recycled MAPHI, there is a certain degree of MA lost, this is corresponded with the decrease in intensity and a small shift.

**Figure S14.** C1s (a), N1s (b) and O1s (c) XPS of the recovered MA^+^-PHI compared with the fresh

**Figure S15.** Powder XRD of MA^+^-PHI before and after catalytic cycling.

**Figure S16.** FT-IR of MA^+^-PHI powder before and after catalytic cycling.

**S.16. TCSPC methodology and data recorded for samples under study**

Time resolved PL spectra were recorded by employing an excitation wavelength of λ_exc_ of 375 nm, while the detection of emission behaviors was done at an λ_em_ of 470 nm. The other operational settings used during spectra acquisition were a laser frequency of 40 MHz and an emission monochromator bandwidth of 2 nm. Decay curves were fitted to a nonlinear equation, lifetime components being determined via the equation:

$$I\left( t \right)=a_{1}\cdot e^{-t/\tau_{1}}+a_{2}\cdot e^{-t/\tau_{2}}$$

***(S8)***

**Figure S17.** PL decay curves obtained for all samples under study (a) and comparison between obtained decay lifetimes recorded for the PHI photocatalysts (b).

**Table S9.** Lifetime components recorded for all PHI materials under study.

| **Sample** | **τ_1_ (ns)** | **τ_2_ (ns)** | **τ_avg_ (ns)** |
| --- | --- | --- | --- |
| ***Na-PHI*** | 3.5 | 0.57 | 2.04 |
| ***H-PHI*** | 5.63 | 1.41 | 3.52 |
| ***MA^+^-PHI*** | 3.49 | 0.03 | 1.76 |

**S.17. In-situ EPR experiments carried out for solid samples**

For carrying out solid EPR experiments, all PHI samples were subjected to an identical routine in which the solid was charged into glass capillaries, while spectra were recorded and after in-situ irradiation with a 415 nm light source (5 minutes).

**Figure S18.** EPR spectra acquired for all PHI samples under dark conditions (a) and after irradiation from a 415 nm light source (b).

**S.18. Methodology and procedures for ionic conductivity measurements and electrochemistry**

Ionic conductivity measurements were performed by use of a four-point probe method. In a typical procedure, 2 g of the PHI materials under investigation were mixed with 0.3mL (0.1 mM) H_3_PO_4_ water solution in the case of proton conductivity, thus achieving a powder texture which allowed the samples to be cold-pressed into rectangular shaped pellets. Compressed specimens were then pressed against the four Pt-probe with a rectangular glass slide, and conductivity measurements were carried out at room temperature.

EIS (electrochemical impedance spectroscopy) tests were conducted on a Gamry Interface 5000E, at an open circuit voltage with a frequency sweep between 0.1 Hz to 100 kHz. Considering the current passes through a 2D finitely extended plate with thickness equals to pellet thickness, the following model was employed ^[39]^:

$$R\boldsymbol{\approx}\frac{D}{\sigma h}$$

***(S9)***

Where h is the thickness of the film (m), D is the center-to-center distance between two parallel electrodes and σ is the bulk conductivity (S/m). Thus, σ is calculated with the geometric parameter of 1.6, and the obtained values are listed in **Table S5**.

**Table S10.** Conductivity values obtained for investigated PHI samples

| **Sample** | **Ionic conductivity (µS·m^-1^)** | **H^+^ conductivity (mS·m^-1^)** |
| --- | --- | --- |
| ***Na-PHI*** | 290 | 4.3 |
| ***H-PHI*** | 360 | 7.46 |
| ***TMA^+^-PHI*** | 93 | 0.37 |
| ***MA^+^-PHI*** | 4400 | 56.8 |
| ***FA^+^-PHI*** | 170 | 14.4 |

**S.19. Determination of VB and CB through VB-XPS and the Tauc method**

**Figure S19.** Tauc plots obtained from processing the DR UV-Vis data of Na-PHI (a), H-PHI (b) and MA^+^-PHI (c). Optical bandgap values are included in the inset of each figure.

**Figure S20.** Valence band energies obtained through XPS data acquisition for Na-PHI (a), H-PHI (b) and MA^+^-PHI (c).

**References**

[1]	A. Rogolino, I. F. Silva, N. V. Tarakina, M. A. R. da Silva, G. F. S. R. Rocha, M. Antonietti, I. F. Teixeira, *ACS Applied Materials & Interfaces* **2022**, *14*, 49820–49829.

[2]	A. Savateev, S. Pronkin, M. G. Willinger, M. Antonietti, D. Dontsova, *Chemistry – An Asian Journal* **2017**, *12*, 1517–1522.

[3]	J. VandeVondele, M. Krack, F. Mohamed, M. Parrinello, T. Chassaing, J. Hutter, *Comput. Phys. Commun.* **2005**, *167*, 103–128.

[4]	G. Lippert, J. Hutter, M. Parrinello, *Mol. Phys.* **1997**, *92*, 477–488.

[5]	T. D. Kühne, *J. Chem. Phys.* **2020**, *152*, 194103.

[6]	J. P. Perdew, K. Burke, M. Ernzerhof, *Phys. Rev. Lett.* **1996**, *77*, 3865–3868.

[7]	S. Grimme, J. Antony, S. Ehrlich, H. Krieg, *J. Chem. Phys.* **2010**, *132*, 154104.

[8]	S. Grimme, S. Ehrlich, L. Goerigk, *J. Comput. Chem.* **2011**, *32*, 1456–1465.

[9]	J. VandeVondele, J. Hutter, *J. Chem. Phys.* **2007**, *127*, 114105.

[10]	S. Goedecker, M. Teter, J. Hutter, *Phys. Rev. B* **1996**, *54*, 1703–1710.

[11]	M. Krack, *Theor. Chem. Acc.* **2005**, *114*, 145–152.

[12]	H. J. Monkhorst, J. D. Pack, *Phys. Rev. B* **1976**, *13*, 5188–5192.

[13]	R. F. W. Bader, *Atoms in Molecules: A Quantum Theory*, Oxford University Press, Oxford, U.K., **1990**.

[14]	A. Otero-de-la-Roza, E. R. Johnson, V. Luaña, *Comput. Phys. Commun.* **2014**, *185*, 1007–1018.

[15]	W. Humphrey, A. Dalke, K. Schulten, *J. Mol. Graph.* **1996**, *14*, 33–38.

[16]	B. O. Community, Blender Foundation, Amsterdam, The Netherlands.

[17]	D. V. Piankova, *Adv. Funct. Mater.* **2025**, *35*, e11389.

[18]	T. D. Kühne, M. Krack, F. R. Mohamed, M. Parrinello, *Phys. Rev. Lett.* **2007**, *98*, 066401.

[19]	T. D. Kühne, *WIREs Comput. Mol. Sci.* **2014**, *4*, 391.

[20]	P. Sharma, T. J. A. Slater, M. Sharma, M. Bowker, C. R. A. Catlow, *Chemistry of Materials* **2022**, *34*, 5511–5521.

[21]	K. S. Schanze, P. V. Kamat, P. Yang, J. Bisquert, *ACS Energy Letters* **2020**, *5*, 2602–2604.

[22]	M. Fang, B. Wang, G. Wan, H. Cao, C. Wang, P. Cao, S. Han, D. Zhu, W. Liu, *Chemical Engineering Journal* **2024**, *494*, 152969.

[23]	W. Lv, H. Cao, J. Zhuo, Y. Cao, H. Liu, M. Wu, T. Yao, L. Sheng, J. Wu, *Journal of Environmental Chemical Engineering* **2025**, *13*, 117791.

[24]	P. Liu, T. Liang, Y. Li, Z. Zhang, Z. Li, J. Bian, L. Jing, *Nature Communications* **2024**, *15*, 53482.

[25]	S. Wu, H. Yu, S. Chen, X. Quan, *ACS Catalysis* **2020**, *10*, 14380–14389.

[26]	X. Zhang, H. Su, P. Cui, Y. Cao, Z. Teng, Q. Zhang, Y. Wang, Y. Feng, R. Feng, J. Hou, X. Zhou, P. Ma, H. Hu, K. Wang, C. Wang, L. Gan, Y. Zhao, Q. Liu, T. Zhang, K. Zheng, *Nature Communications* **2023**, *14*, 42887.

[27]	X. Chen, L. Nie, X. Li, *Chemistry – An Asian Journal* **2025**, *20*.

[28]	C. M. Pelicano, J. Li, M. Cabrero-Antonino, I. F. Silva, L. Peng, N. V. Tarakina, S. Navalón, H. García, M. Antonietti, *Journal of Materials Chemistry A* **2024**, *12*, 475–482.

[29]	A. Galushchinskiy, C. Pulignani, H. Szalad, E. Reisner, J. Albero, N. V. Tarakina, C. M. Pelicano, H. Garcia, O. Savateev, M. Antonietti, *Solar RRL* **2023**, *7*.

[30]	H. Szalad, A. Galushchinskiy, T. Jianu, V. Roddatis, N. V. Tarakina, O. Savateev, M. Antonietti, J. Albero, H. Garcia, *Applied Catalysis B: Environmental & Energy* **2024**, *357*, 124323.

[31]	H. Tong, J. Odutola, J. Song, L. Peng, N. Tkachenko, M. Antonietti, C. M. Pelicano, *Advanced Materials* **2024**, *36*, 2412753.

[32]	Y. Li, Y. Guo, G. Fan, D. Luan, X. Gu, X. W. Lou, *Angew Chem Int Ed* **2024**, *63*.

[33]	C. Qiao, W. Xian, Z. Lai, Z. Huang, Q. Guo, S. Wang, Z. Dai, Y. Xiong, X. Meng, S. Ma, Q. Sun, *Angew Chem Int Ed* **2025**, *64*.

[34]	J. Sun, H. Sekhar Jena, C. Krishnaraj, K. Singh Rawat, S. Abednatanzi, J. Chakraborty, A. Laemont, W. Liu, H. Chen, Y.-Y. Liu, K. Leus, H. Vrielinck, V. Van Speybroeck, P. Van Der Voort, *Angewandte Chemie International Edition* **2023**, *62*, e202216719.

[35]	W. Wu, Z. Li, S. Liu, D. Zhang, B. Cai, Y. Liang, M. Wu, Y. Liao, X. Zhao, *Angew Chem Int Ed* **2024**, *63*.

[36]	J. Bharti, J. Odutola, Z. Hajiahmadi, K. Nolkemper, Z. Tian, H. Tong, V. Shvalagin, T. D. Kühne, T.-P. Ruoko, C. M. Pelicano, *Adv. Mater.* **2026**, 38, e10585.

[37]	X. Fang, J. Huang, Z. Zhang, M. Wu, S. Ma, Y. Gao, X. Yang, Y. Li, Y. Zhu, Y. Ding, *ACS Nano* **2026**, 20, 14847.

[38]	X. Fang, B. Li, J. Huang, C. Hu, X. Yang, P. Feng, X. Dong, J. Wu, Y. Li, Y. Ding, *Energy Environ. Sci.* **2025**, 18, 6202.

[39]	T. M. Nahir, *Journal of the American Chemical Society* **2005**, *127*, 12431–12431.
